# Spectroscopic and Theoretical Insights Into High‐Entropy‐Alloy Surfaces and Their Interfaces with Semiconductors for Enhanced Photocatalytic Hydrogen Production

**DOI:** 10.1002/smll.202503512

**Published:** 2025-05-08

**Authors:** Jui‐Tai Lin, Yueh‐Chun Hsiao, Chao Li, Ching‐Yuan Tseng, Zong‐Ying He, Adrian M. Gardner, Yi Chen, Chueh‐Cheng Yang, Chia‐Hsin Wang, Shang‐Cheng Lin, Xin‐Xuan Lin, Chih‐Yi Lin, Kun‐Han Lin, Alexander J. Cowan, Tung‐Han Yang

**Affiliations:** ^1^ Department of Chemical Engineering National Tsing Hua University Hsinchu 300044 Taiwan; ^2^ Stephenson Institute for Renewable Energy and Department of Chemistry University of Liverpool Liverpool L69 7ZF UK; ^3^ Department of Chemistry The Hong Kong University of Science and Technology Kowloon (SAR) Hong Kong 999077 China; ^4^ College of Semiconductor Research National Tsing Hua University Hsinchu 300044 Taiwan; ^5^ Early Career Laser Laboratory University of Liverpool Liverpool L69 3BX UK; ^6^ National Synchrotron Radiation Research Center Hsinchu 300092 Taiwan; ^7^ High Entropy Materials Center National Tsing Hua University Hsinchu 300044 Taiwan

**Keywords:** density functional theory (DFT), high‐entropy alloy, in situ X‐ray photoelectron spectroscopy, photocatalytic hydrogen production, transient absorption spectroscopies

## Abstract

Recently, high‐entropy alloy (HEA) nanocatalysts have shown outstanding catalytic performance. However, their integration with semiconductors for photocatalytic reactions remains largely unexplored. Here, Pd@HEA core–shell nanocrystals with controlled compositions and facets on TiO_2_ supports are synthesized, achieving significantly enhanced photocatalytic hydrogen production. Compared to Pd@Pt/TiO_2_, Pd@Pt_0.4_Pd_0.15_Ir_0.15_Ru_0.15_Rh_0.15_ core–shell nanocubes/TiO_2_ exhibit superior photoactivity, driven by optimized Schottky junctions and synergistic multimetallic interactions that enhance photocatalysis. UV photoelectron spectroscopy reveals a high work function of 4.81 eV for Pd@Pt_0.4_Pd_0.15_Ir_0.15_Ru_0.15_Rh_0.15_, enabling efficient charge separation between Pd@HEA and TiO₂. Meanwhile, transient absorption spectroscopy confirms a significantly prolonged carrier lifetime of 4 ms, far surpassing that of pure TiO_2_; (65 µs). In addition, in situ X‐ray photoelectron spectroscopy confirms that photo‐induced electrons preferentially accumulate on Ir and Pt sites, increasing their electron density and identifying them as primary adsorption sites. Furthermore, density functional theory calculations further reveal that Pt‐based bridge sites exhibit a more optimal hydrogen binding free energy than Ir‐based sites, suggesting that Pt serves as the dominant active site in photocatalysis. This study establishes a framework for the rational design of HEA‐semiconductor photocatalysts, providing fundamental insights for solar‐driven hydrogen production.

## Graphical abstract

High‐entropy‐alloy‐based nanocrystals on semiconductors enhance photocatalytic hydrogen production through optimized Schottky junctions and multimetallic synergy, as revealed by spectroscopic and theoretical insights. Ultraviolet photoelectron and transient absorption spectroscopy confirm efficient charge separation and extended carrier lifetime, while in situ X‐ray photoelectron spectroscopy and density functional theory identify platinum as the dominant active site for hydrogen adsorption and catalysis.

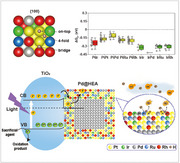

## 1 Introduction

Photocatalytic hydrogen production with metal–semiconductor hybrid photocatalysts is widely considered one of the most promising and sustainable strategies for hydrogen production from solar energy. In general, metals can act as electron sinks, reducing the recombination of photo‐generated electron–hole pairs in the semiconductor, thereby increasing the efficiency of charge separation and prolonging carrier lifetimes.^[^ ^1^, ^2^, ^3^ ^]^ As a result, the overall photocatalytic performance is improved. Over the past two decades, various metals like platinum‐group metals (PGMs: Pt, Pd, Ir, Ru, and Rh) have frequently been used as co‐catalysts due to their large work functions, active surfaces, and excellent stability under light irradiation.^[^ ^4^, ^5^, ^6^ ^]^ However, a significant challenge arises in achieving a balance between optimal Schottky junction and highly active sites for photocatalytic hydrogen production, thereby hindering further enhancement of photocatalytic performance.^[^ ^7^, ^8^ ^]^ For example, certain metals that form a suitable Schottky junction with semiconductors to effectively separate electron–hole pairs may not necessarily offer a suitable surface for photocatalytic reactions, and vice versa. Bimetallic and multimetallic alloy co‐catalysts may address this challenge. By adjusting their compositions, the work function can be fine‐tuned to establish an optimal Schottky barrier between the semiconductor and metal. In addition, the synergistic interactions between different elements of the multimetallic catalysts can be leveraged, allowing for the creation of a surface with active sites where hydrogen adsorption and desorption are neither too strong nor too weak. As a result, alloy co‐catalysts are expected to effectively achieve an optimal Schottky junction along with high surface activity, thereby enhancing overall photocatalytic performance.^[^ ^8^ ^]^

Recently, high‐entropy alloy (HEA) nanocatalysts, composed of at least five different elements, have demonstrated outstanding catalytic performance in various electrocatalytic and thermal catalytic reactions.^[^ ^9^, ^10^ ^]^ These HEA nanocatalysts surpass the capabilities of traditional monometallic and bimetallic catalysts. The unique synergy between the multiple elements, often termed the “cocktail effect,” enables the creation of a wide array of active sites with optimal binding energies for reactants, intermediates, and products.^[^ ^11^, ^12^, ^13^ ^]^ For instance, both theoretical calculations and experimental studies have revealed that multi‐elemental HEA nanocrystals such as PtPdIrRuRh,^[^ ^14^, ^15^, ^16^ ^]^ PtRuFeCoNi,^[^ ^17^ ^]^ PtRhFeCoNi,^[^ ^18^ ^]^ RuMnFeCoNi,^[^ ^19^ ^]^ PtRuNiCoFeMo,^[^ ^20^ ^]^ and PtPdIrRuRhOsAgAu^[^ ^21^ ^]^ exhibited optimized binding energies for intermediates like H^*^ and OH^*^, facilitating improved activity in hydrogen production and oxidation reactions, in accordance with the Sabatier principle. Additionally, the nearly infinite chemical space in HEA catalysts enables extensive tunability and optimization of their catalytic properties, offering a vast landscape for designing catalysts with tailored functionalities. These traits suggest that HEA catalysts could potentially improve charge separation when integrated with semiconductor materials, thus boosting overall photocatalytic activity. Along these lines, a pressing challenge is how to not only fine‐tune the work function of HEA nanocrystals to achieve optimal Schottky junctions but also ensure a large number of active sites. Addressing this challenge involves developing strategies to precisely control the compositions and surface atomic arrangement of HEA nanocrystals, thereby optimizing their electronic properties and catalytic activities for enhanced photocatalytic performance. Most importantly, a knowledge gap exists in understanding the band structure and charge carrier dynamics of HEA‐semiconductor hybrids under light irradiation. Furthermore, a comprehensive understanding of the synergistic effects within high‐entropy multi‐elemental surfaces and their true active sites is essential. In situ characterization tools, combined with theoretical calculations, could provide these insights, opening up new opportunities for the rational design of HEA‐semiconductor photocatalysts with strong atomic‐scale synergistic effects.

Despite the significant advancements in electrocatalytic and thermal catalytic applications, the use of HEA catalysts in photocatalysis remains in its early stages of research.^[^ ^15^, ^22^, ^23^, ^24^ ^]^ A summary of recent reports on HEA‐semiconductor photocatalysis is provided in Table S1 (Supporting Information). For example, a representative study demonstrated that Pt_18_Cu_27_Fe_15_Co_14_Ni_26_ spherical HEA nanoparticles combined with protonated g‐C_3_N_4_ nanosheets exhibited remarkable performance in photocatalytic hydrogen production.^[^ ^25^ ^]^ While these pioneering studies have reported HEA‐semiconductor hybrid systems for photocatalytic applications, most have primarily focused on material synthesis and overall catalytic performance, without systematically examining how compositional tuning and facet control of HEA surfaces influence photocatalytic behavior. Of particular note is that we carried out a preliminary study of a single composition of core–shell HEA/TiO_2_ photocatalysts.^[^ ^15^ ^]^ These showed exceptional activity, but the system was not further optimized. In addition, the mechanistic understanding of HEA surfaces and their interfaces with semiconductors for enhanced photocatalytic hydrogen production remains largely underexplored.

In the present study, we conduct a comprehensive investigation into how variations in HEA composition and surface facets impact the photocatalytic properties of HEA‐semiconductor hybrids. We demonstrate that HEA nanocrystals serve as ideal candidates for simultaneously optimizing the Schottky junction and enhancing the surface reactivity, thereby enabling superior photocatalytic performance. We synthesized a series of quinary Pd@PGM‐HEA core–shell nanocrystals with controlled compositions and facets using a heteroepitaxial growth in the dropwise synthesis. These Pd@PGM‐HEA nanocrystals were then dispersed as co‐catalysts on commercial TiO_2_ (Degussa P25) for photocatalytic hydrogen production. By systematically varying the compositions and facets of the Pd@PGM‐HEA nanocrystals, we observed a significant enhancement in photocatalytic performance compared to Pd@Pt nanocrystals as co‐catalysts. The superior activity of the Pd@PGM‐HEA nanocrystals was attributed to their ability to form optimal Schottky junctions with TiO_2_, as well as the synergistic interactions between the constituent elements. UV photoelectron spectroscopy (UPS) and transient absorption spectroscopy (TAS) were employed to probe the band structure and charge carrier dynamics of the HEA‐semiconductor hybrids, revealing that the highly tunable Pd@PGM‐HEA nanocrystals outperformed Pt‐based nanocrystals in extending carrier lifetimes. Furthermore, in situ, XPS and density functional theory (DFT) identified Pt‐enriched sites as the primary adsorption and active sites on PGM‐HEA surfaces during photocatalytic operation. Together, the formation of a higher Schottky barrier and the presence of a Pt‐enriched catalytically favorable surface work synergistically to enhance photocatalytic performance. This research underscores the immense potential of HEA‐semiconductor hybrids in photocatalytic applications, which can develop next‐generation photocatalysts that offer unprecedented efficiency and performance in solar energy conversion.

## 2 Results and Discussion

### 2.1 Synthesis of Pd@PtPdIrRuRh Core–Shell Nanocrystals with Controlled Compositions and Facets on TiO_2_

**Figure 1:**
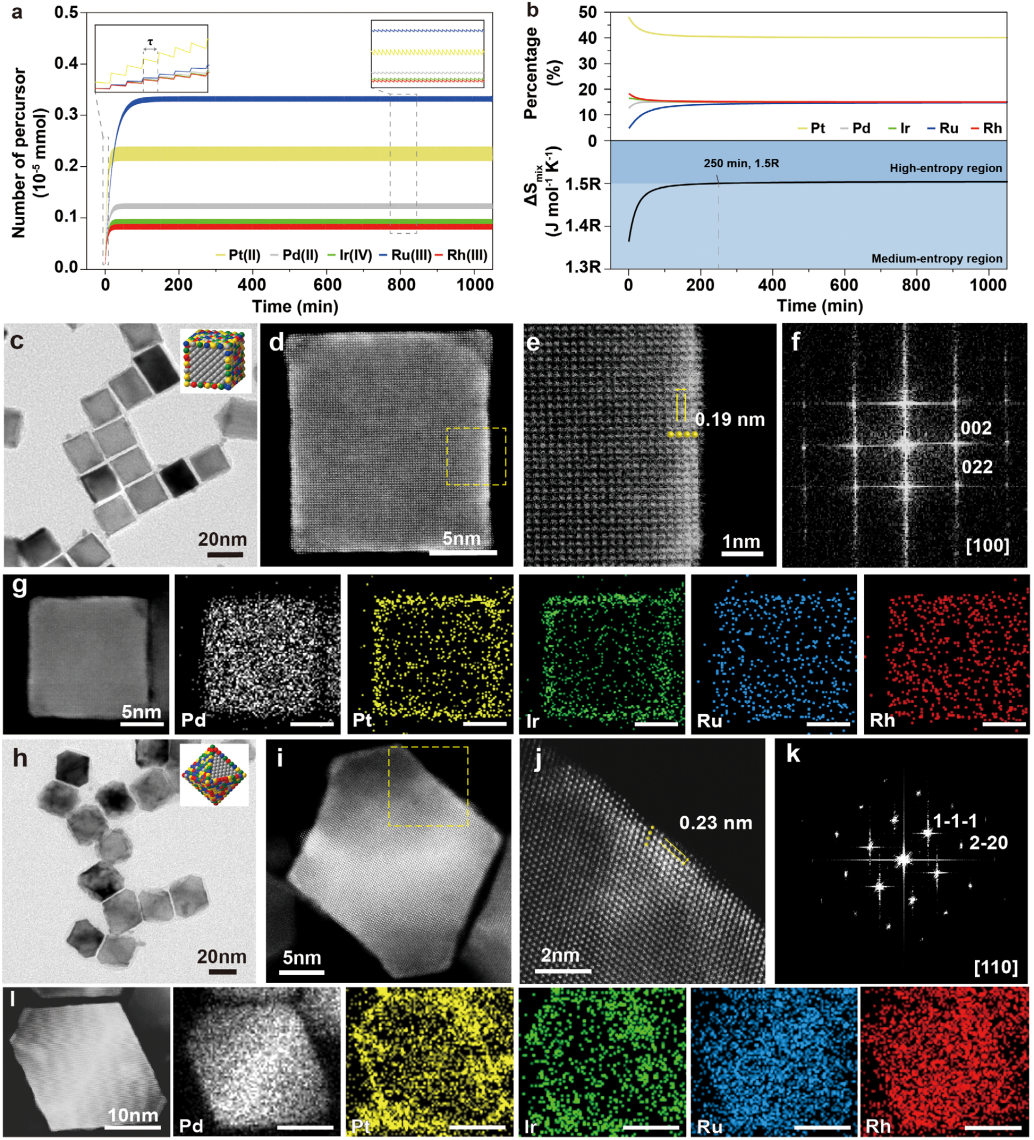
Materials synthesis and characterizations of Pd@HEA core–shell nanocubes and octahedra. a) The simulated numbers of Pt(II), Pd(II), Ir(IV), Ru(III), and Rh(III) precursor ions remaining in the reaction solution, b) the simulated instantaneous percentages of deposited Pt, Pd, Ir, Ru, and Rh atoms. c) TEM (insert is an atomic model of Pd@HEA core–shell nanocubes), d,e) HAADF‐STEM images, f) FFT pattern of e,g) EDS mappings of Pd@Pt_0.4_Pd_0.15_Ir_0.15_Ru_0.15_Rh_0.15‐4L_ core–shell nanocubes. h) TEM (insert is an atomic model of Pd@HEA core–shell octahedra), i,j) HAADF‐STEM images, and k) FFT pattern of j,l) EDS mappings of Pd@Pt_0.4_Pd_0.15_Ir_0.15_Ru_0.15_Rh_0.15‐4L_ core–shell octahedra.

In this study, we successfully synthesized multimetallic RuRhPdPtIr shells with approximately four atomic layers on well‐defined Pd seeds through heteroepitaxial growth using a dropwise synthesis approach based on our recent report.^[^ ^15^ ^]^ This method allowed precise control over the composition and facets of the Pd@RuRhPdPtIr core–shell nanocrystals. In a standard synthesis procedure, a precursor solution comprising five PGM salt precursors (Pd(II), Pt(II), Ir(III), Ru(III), and Rh(II)) was gradually introduced at a controlled rate of 0.8 mL h^−1^ into a preheated ethylene glycol (EG) solution. This solution contained pre‐synthesized Pd seeds with cubic or octahedral shapes, *L*‐ascorbic acid (AA) as the reducing agent, poly(vinylpyrrolidone) (PVP) as a stabilizing agent, and KBr to slow down the reduction process, all maintained at 195 °C (Figure S1, Supporting Information). In general, the growth behavior of metal shells on nanocrystal seeds is primarily governed by the relative rates of atomic deposition from precursor reduction and atomic diffusion across the seed surface. When the surface diffusion rate of adatoms surpasses the rate at which new atoms are deposited, the system favors a layer‐by‐layer growth mode.^[^ ^26^, ^27^ ^]^ In this case, atoms are able to migrate across the surface and occupy energetically favorable positions, resulting in smooth, epitaxial layers that conform to the facets of seeds. Conversely, if the deposition rate significantly exceeds the diffusion rate, atoms tend to remain at their initial landing sites, leading to local accumulation and the emergence of island growth mode These competing dynamics are influenced by several parameters, including reaction temperature, and injection rate, which collectively shape the morphology and interface quality of the resulting core–shell nanocrystals. In our study, the combination of a slow precursor injection rate, which ensured a low rate of atomic deposition, and an elevated reaction temperature, which promoted rapid surface diffusion, created favorable conditions for layer‐by‐layer epitaxial growth. Under these conditions, the five PGM elements were uniformly deposited onto the preformed cubic or octahedral Pd seeds, yielding atomically smooth PtPdIrRuRh shells bounded by well‐defined {100} or {111} facets, respectively. Importantly, by varying the ratios of the five PGM salt precursors, we prepared multimetallic PtPdIrRuRh shells with six different ratio compositions, adjusting the Pt atomic ratios from 0%, 20%, 40%, 60%, 80%, to 100% while maintaining equimolar amounts of the other four PGMs (i.e., Pd@Pd_0.25_Ir_0.25_Ru_0.25_Rh_0.25‐4L_, Pd@Pt_0.2_Pd_0.2_Ir_0.2_Ru_0.2_Rh_0.2‐4L_, Pd@Pt_0.4_Pd_0.15_Ir_0.15_Ru_0.15_Rh_0.15‐4L_, Pd@Pt_0.6_Pd_0.1_Ir_0.1_Ru_0.1_Rh_0.1‐4L_, Pd@Pt_0.8_Pd_0.05_Ir_0.05_Ru_0.05_Rh_0.05‐4L_, and Pd@Pt_4L_, as shown in **Figures** **1** and S2 (Supporting Information). This adjustment in Pt ratio is based on the recognition that Pt is widely regarded as the most effective co‐catalyst for photocatalytic hydrogen production.^[^ ^28^ ^]^

The dropwise addition of mixed metal precursors enables control over the steady‐state concentration of each precursor during the epitaxial growth process, ensuring that atoms from different precursors are introduced consistently. This approach facilitates the simultaneous deposition of metal atoms in desired atomic ratios, thus promoting the uniform formation of atomic mixing in HEA layers. As illustrated in Figure 1a, we quantified the concentration of each precursor remaining in the reaction medium during the epitaxial growth of Pd@Pt_0.4_Pd_0.15_Ir_0.15_Ru_0.15_Rh_0.15‐4L_ core–shell nanocubes, which consist of four atomic layers of the HEA shell. This estimation was derived through a combination of kinetic experiments, where we measured the reduction rate constants of the precursors, and mathematical modeling (detailed in the Experimental Section). The Rh(III), Pt(II), Ir(IV), Pd(II), and Ru(III) precursors quickly reach equilibrium within ≈22, 27, 24, 43, and 170 min respectively, once the dropwise addition commences. This suggests that the five metals are generated from the metal precursors in a controlled atomic ratio (Pt: Pd: Ir: Ru: Rh = 0.4: 0.15: 0.15: 0.15: 0.15) during a prolonged period of steady‐state growth. Additionally, Figure 1b presents the calculated instantaneous percentages of formed metal atoms during the growth process. The results indicate that, after a brief initial stage, the proportions of the five PGM elements remain nearly constant. Furthermore, the instantaneous entropy of mixing (Δ*S*_mix_) was computed using the formula $\Delta S_{\mathrm{mix}}=-R\sum_{i\;=1}^{n}x_{i}\ln x_{i}$, where *R* is the gas constant and x_i_ represents the atomic fraction of component i. As shown in Figure 1b, the entropy of mixing reaches 1.5 *R* after 250 min reaction, which meets the definition of high‐entropy materials, where the entropy of mixing is typically greater than 1.5 *R*. This enhanced entropy likely facilitates the alloying of the metal atoms, driving the formation of the HEA layers during solution‐phase epitaxial growth.

Figure 1c–g shows the material characterizations of Pd@Pt_0.4_Pd_0.15_Ir_0.15_Ru_0.15_Rh_0.15‐4L_ core–shell nanocubes. The transmission electron microscopy (TEM) image (Figure 1c) reveals that Pd@Pt_0.4_Pd_0.15_Ir_0.15_Ru_0.15_Rh_0.15‐4L_ maintains their cubic shape after the deposition of the five PGM atoms onto the cubic seeds, suggesting successful facet engineering with no significant self‐nucleation events observed. This also implies that the growth process was well‐controlled, ensuring that the atoms were deposited uniformly onto the pre‐formed cubic seeds. Atomic‐resolution high‐angle annular dark‐field scanning transmission electron microscopy (HAADF‐STEM) images (Figure 1d,e) reveal the contrast between the Pd cores and the HEA shells, attributed to the difference in atomic numbers. The HAADF‐STEM images show that the average thickness of the deposited Pt_0.4_Pd_0.15_Ir_0.15_Ru_0.15_Rh_0.15‐4L_ layers is approximately four atomic layers. This contrast underscores the clear distinction between the core and shell regions, highlighting the successful and uniform deposition of the HEA layers. The average lattice fringe spacing is measured at ≈0.19 nm, which corresponds to the distance between the (200) plane of the face‐centered cubic (FCC) HEA structure. The fast Fourier transform (FFT) diffractogram (Figure 1f) further confirms a single set of FCC diffraction patterns along the [100] zone axis, indicating the successful epitaxial growth of HEA shells on the cubic seeds. These results are consistent with the XRD patterns of both pure Pd and Pd@Pt_0.4_Pd_0.15_Ir_0.15_Ru_0.15_Rh_0.15‐4L_ core–shell nanocubes, which also exhibit characteristic reflections of an FCC structure without any evidence of impurity phases or phase segregation (Figure S4, Supporting Information). Energy‐dispersive spectroscopy (EDS) maps (Figure 1g) confirm that Pt, Pd, Ir, Ru, and Rh atoms are uniformly distributed throughout the four‐layer shells, with no significant element segregation despite the higher Pt proportion. The atomic percentage of the five PGMs was further analyzed by inductively coupled plasma optical emission spectrometry (ICP‐OES), confirming that the atomic proportions of Pt, Pd, Ir, Ru, and Rh in the shells are 37.2%, 15.7%, 11.2%, 19.7%, and 16.3%, respectively. This composition is consistent with the original feed ratios of the PGM precursors (Table S2, Supporting Information).

In addition, facet engineering of Pt_0.4_Pd_0.15_Ir_0.15_Ru_0.15_Rh_0.15‐4L_ was applied to octahedral Pd seeds enclosed by {111} facets, as shown in Figure 1h–k. The resulting Pd@Pt_0.4_Pd_0.15_Ir_0.15_Ru_0.15_Rh_0.15‐4L_ core–shell octahedra maintained their octahedral shape, though the surface exhibited slight roughness (Figure 1i). Additionally, the deposition of HEA atomic layers occurred preferentially on the {100} facets rather than the {111} surfaces, as thicker HEA atomic layers were observed on the {100} facets (Figure S3, Supporting Information). This could be due to the higher surface energy of the {100} facets compared to {111}.^[^ ^29^ ^]^ As shown in Figure 1h–k, the atomic‐resolution HAADF‐STEM images, and the corresponding FFT diffractogram indicate that the HEA atomic layers are primarily enclosed by (111) facets, with a thickness of about four atomic layers and lattice fringes measuring 0.23 nm. EDS maps (Figure 1l) of the Pd@Pt_0.4_Pd_0.15_Ir_0.15_Ru_0.15_Rh_0.15‐4L_ core–shell octahedra confirmed that the deposited shells are composed of the five PGM elements. ICP‐OES analysis (Table S2, Supporting Information) further revealed their atomic proportions within the shells: Pt (35%), Pd (16.2%), Ir (15.9%), Ru (18.5%), and Rh (14.3%). These results confirm the successful incorporation of the alloy components in the core–shell structure.

**Figure 2:**
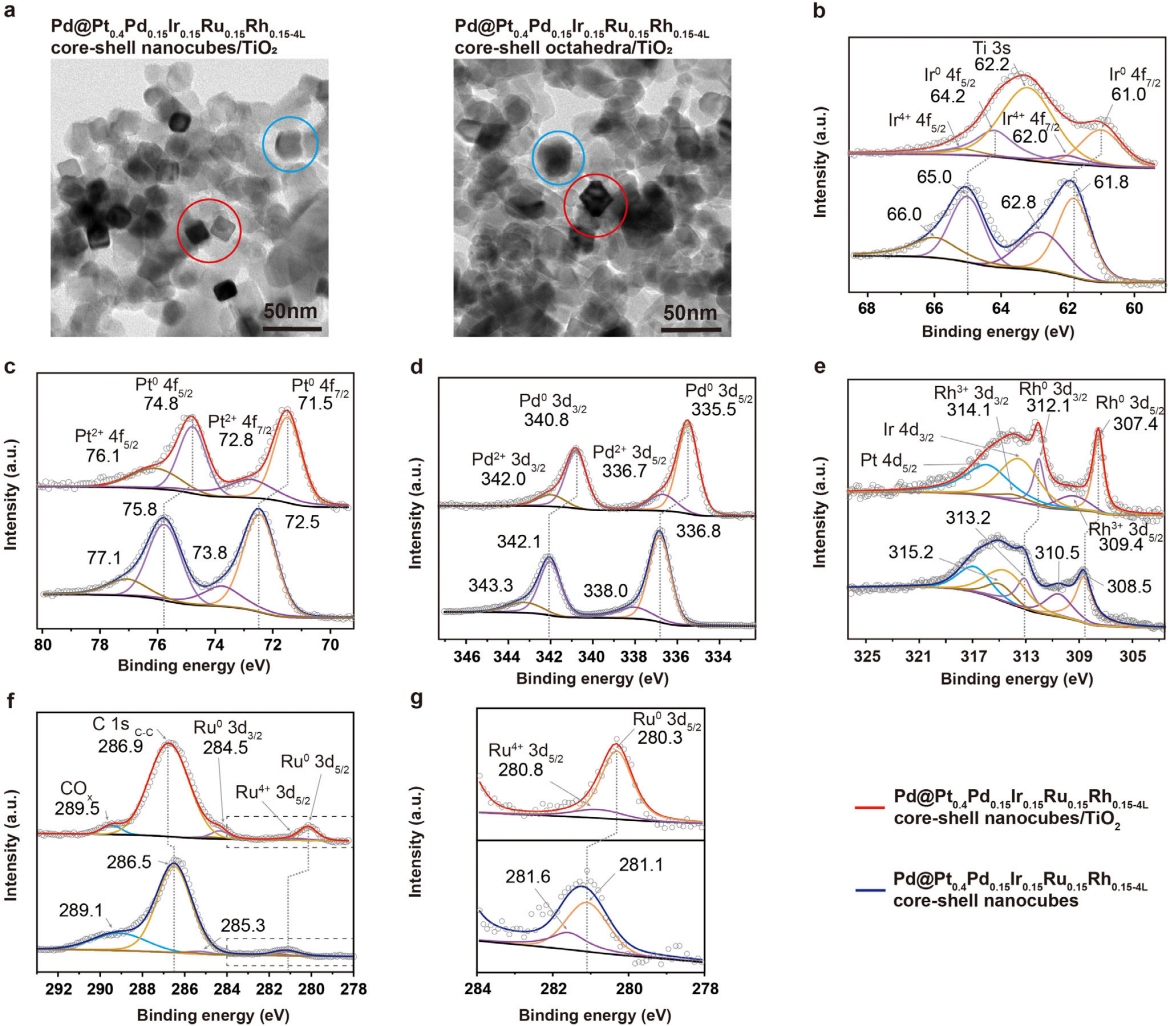
Surface chemical states of Pd@HEA core–shell nanocrystals on TiO_2_. a) TEM images of Pd@Pt_0.4_Pd_0.15_Ir_0.15_Ru_0.15_Rh_0.15‐4L_ core–shell nanocubes and octahedra (marked by red circles) dispersed on the TiO_2_ supports (marked by blue circles) through the heat treatments. b–g) XPS spectra of b) Ti 3s and Ir 4f, c) Pt 4f, d) Pd 3d, e) Pt 4d, Ir 4d, Rh 3d, f) C 1s and g) Ru 3d for Pd@Pt_0.4_Pd_0.15_Ir_0.15_Ru_0.15_Rh_0.15‐4L_ core–shell nanocubes/TiO_2_ (red lines) and pure Pd@Pt_0.4_Pd_0.15_Ir_0.15_Ru_0.15_Rh_0.15‐4L_ core–shell nanocubes (blue lines).

Furthermore, the Pd@PtPdIrRhRu core–shell nanocrystals were dispersed onto TiO_2_ supports using an ultrasonic method for 0.5 h, followed by annealing at 190 °C in the air for 2 h and then in a 10% H_2_/N_2_ environment for 1 h. This annealing process was conducted to eliminate any organic compounds adsorbed on the surface and to improve the interaction between the Pd@PtPdIrRuRh nanocrystals and the TiO_2_ semiconductor, ensuring better contact and promoting more effective catalytic performance. A reducing environment is required to avoid excessive formation of the oxide state of the HEA. The resulting metal–semiconductor hybrid photocatalysts, containing 5 wt.% Pd@PtPdIrRhRu were then tested for photocatalytic hydrogen production. As shown in **Figure** **2**a, the Pd@Pt_0.4_Pd_0.15_Ir_0.15_Ru_0.15_Rh_0.15‐4L_ core–shell nanocubes, and octahedra were uniformly dispersed on the TiO_2_ supports, and their shapes were well maintained after the heat treatments. Moreover, XRD analysis (Figure S4, Supporting Information) further confirmed that the FCC phase remained unchanged, providing strong evidence for the phase purity and structural integrity of the Pd@HEA nanocrystals and their hybrids with TiO_2_.

In addition, the XPS spectra in Figure 2b–f show the surface chemical states of the representative Pt_0.4_Pd_0.15_Ir_0.15_Ru_0.15_Rh_0.15‐4L_ core–shell nanocubes and the electronic interactions between the Pt_0.4_Pd_0.15_Ir_0.15_Ru_0.15_Rh_0.15‐4L_ core–shell nanocubes and the TiO_2_ support following heat treatments, with detailed peak positions marked on each spectrum through curve fitting (integrated into Table S3, Supporting Information). It can be observed that the mixed elements in Pt_0.4_Pd_0.15_Ir_0.15_Ru_0.15_Rh_0.15‐4L_ are primarily in the metallic state, with a minor fraction in the oxidized state.^[^ ^30^ ^]^ This assessment is based on the peak positions and the area ratios of metallic and oxidized states derived from the curve fitting results. Importantly, compared to the pure Pt_0.4_Pd_0.15_Ir_0.15_Ru_0.15_Rh_0.15‐4L_, the decrease in binding energies for Pt, Pd, Rh, Ir, and Ru in the Pt_0.4_Pd_0.15_Ir_0.15_Ru_0.15_Rh_0.15‐4L_ on TiO_2_ support after heat treatment indicates that electron transfer occurs from the TiO_2_ support to the HEA surface. This shift in binding energies implies the electronic interactions between the TiO_2_ and the HEA. As shown in Figure 2b, the Ir 4f and Ti 3s peaks overlap due to their proximity in binding energy. By utilizing curve fitting, the Ir 4f_7/2_ and 4f_5/2_ peaks were successfully separated from the Ti 3s peak, allowing for a precise analysis of the binding energy shifts. The metallic 4f_7/2_ and 4f_5/2_ Ir^0^ peaks shifted from ≈61.8 and 65 eV to lower binding energies, ≈61 eV and 64.2 eV, respectively, after the formation of the Pt_0.4_Pd_0.15_Ir_0.15_Ru_0.15_Rh_0.15‐4L_/TiO_2_ hybrid structure. Similarly, a noticeable shift in the binding energies was also observed in the Pt 4f and Pd 3d peaks, reflecting a similar trend, as shown in Figure 2c,d. In particular, we further demonstrate the use of curve fitting to successfully resolve the overlapping peaks of Rh, Pt, and Ir in the range of 325–305 eV. The Rh 3d peaks were deconvoluted into their metallic (Rh^0^) and oxidized (Rh^3+^) states, as shown in Figure 2e. Again, the primary metallic Rh 3d_5/2_ and Rh 3d_3/2_ peaks shifted from ≈308.5 and 313.2 eV to lower binding energies of 307.4 and 312.1 eV, respectively. In the range of 292–278 eV, the Ru 3d and C 1s peaks were observed to overlap, as shown in Figure 2f. Based on the curve fitting, the Ru 3d XPS spectrum (inset of Figure 2f) revealed the coexistence of major metallic (Ru^0^) and minor oxidized (Ru^4+^) species. Both species exhibited a shift to smaller binding energies after the heat treatment. For C 1s, the signals can be distinguished into C─C and CO_x_ species, likely originating from adsorbed PVP on the surface and/or organic contaminants from the air.^[^ ^31^ ^]^ Importantly, after heat treatment, the peak area ratio of C 1s to Ru 3d decreased, and the peak corresponding to CO_x_ species became smaller. This suggests that part of the PVP and/or organic contaminants were removed from the surface during the heat treatment, resulting in a cleaner surface with fewer organic residues. The XPS spectra of the Pt_0.4_Pd_0.15_Ir_0.15_Ru_0.15_Rh_0.15‐4L_ core–shell nanocubes/TiO_2_ were recorded after undergoing heat treatments without exposure to a 10% H_2_/N_2_ reducing atmosphere for 1 h (instead annealed solely in air for 2 h) and the TiO_2_ support underwent different heat treatment stage were presented in Figure S5 (Supporting Information), these findings underscore the essential role of a reducing environment in achieving a clean metallic‐state catalyst surface, as it leads to a decrease in the binding energies of the five noble metal components.^[^ ^32^ ^]^

### 2.2 Photocatalytic Hydrogen Production

The hydrogen production activity in photocatalytic hydrogen production was evaluated using a 10 mL aqueous solution containing 0.4 M AA as a sacrificial agent under Xenon light irradiation with a solar spectral correction filter (simulated sunlight irradiation, 1000 W m^−2^, *λ* > 300 nm). The amount of evolved hydrogen was quantified by gas chromatography (GC). Detailed experimental procedures are provided in the Experimental Section. The use of 1 mg of photocatalyst dispersed in 10 mL of deionized water was selected as a control condition by fixing the TiO_2_ amount at 1 mg, allowing us to systematically evaluate the performance of the photocatalyst under varying HEA loadings. The total amount of the HEA/TiO_2_ photocatalyst significantly influences photocatalytic activity, with an optimal concentration range. Exceeding this range results in a decline in activity, primarily due to excessive TiO_2_ in the solution obstructing light penetration.^[^ ^33^ ^]^ Since the focus of this work is to investigate the facet‐ and composition‐dependent photocatalytic behavior of HEAs, we fixed the TiO_2_ loading at a small amount of 1 mg per 10 mL solution in all experiments. This low and consistent setting ensures reliable comparisons while minimizing the consumption of noble metals. To further validate this approach, we conducted a systematic optimization of HEA loading using Pd@Pt_0.2_Pd_0.2_Ir_0.2_Ru_0.2_Rh_0.2‐4L_ core–shell nanocubes supported on TiO_2_ (Figure S6, Supporting Information). When only 0.025 mg of HEA was used, the hydrogen production rate was minimal. Increasing the loading to 0.05 mg significantly enhanced performance. However, further increasing the amount to 0.075 mg resulted in a decline, likely due to excessive coverage of the TiO_2_ surface, which limited light penetration and reduced the effective interface between TiO_2_ and the co‐catalyst. These results confirm that an optimal loading under the tested conditions is 0.05 mg of HEA per 1 mg of TiO_2_.

**Figure 3:**
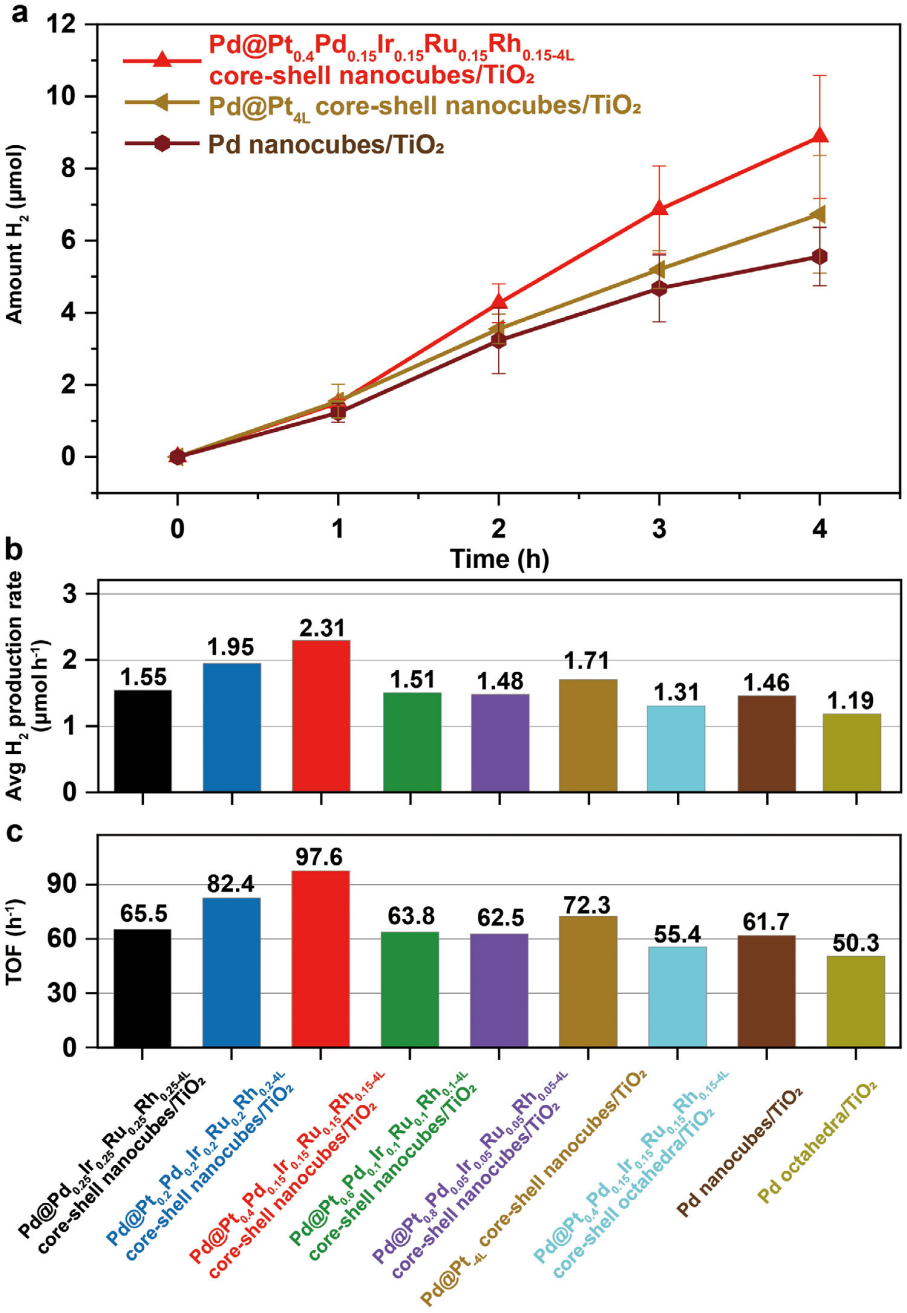
Photocatalytic hydrogen production. a) Photocatalytic hydrogen production performance of three representative catalysts including Pd@Pt_0.4_Pd_0.15_Ir_0.15_Ru_0.15_Rh_0.15‐4L_ core–shell nanocubes/TiO_2_, Pd@Pt_4L_ core–shell nanocubes/TiO_2_, and Pd nanocubes/TiO_2_ photocatalysts under simulated sunlight irradiation (*λ* > 300 nm) over 4 h. b) Average hydrogen production rate over the 4‐hour duration and c) turnover frequency (TOF) comparison for various catalysts evaluated in this study.

To explore the impacts of composition on photocatalytic performance, the Pt content was systematically varied while maintaining equimolar concentrations of the other four PGMs in the multi‐component surface. These alloyed surfaces were deposited on the Pd nanocubes. This approach yielded six core–shell nanocubes with {100} facets, then supported on TiO_2_ as co‐catalysts: Pd@Pd_0.25_Ir_0.25_Ru_0.25_Rh_0.25‐4L_, Pd@Pt_0.2_Pd_0.2_Ir_0.2_Ru_0.2_Rh_0.2‐4L_, Pd@Pt_0.4_Pd_0.15_Ir_0.15_Ru_0.15_Rh_0.15‐4L_, Pd@Pt_0.6_Pd_0.1_Ir_0.1_Ru_0.1_Rh_0.1‐4L_, Pd@Pt_0.8_Pd_0.05_Ir_0.05_Ru_0.05_Rh_0.05‐4L_, and Pd@Pt_4L_. Additionally, we tested the photocatalytic performance of Pd@Pt_0.4_Pd_0.15_Ir_0.15_Ru_0.15_Rh_0.15‐4L_ core–shell octahedra with {111} facets supported on TiO_2_ to investigate the facet effect. The photocatalytic activity of pure TiO_2_ was also evaluated as a control sample, showing no detectable hydrogen production as determined by GC measurements (Figure S6, Supporting Information).^[^ ^34^, ^35^ ^]^ Among all the photocatalysts, the Pd@Pt_0.4_Pd_0.15_Ir_0.15_Ru_0.15_Rh_0.15‐4L_ core–shell nanocubes/TiO_2_ exhibited the highest catalytic activity, achieving a hydrogen production rate of 2.31  ±  0.46 µmol h^−1^ (**Figure** **3**a,b). This activity was notably 1.4 times higher than that of the Pd@Pt_4L_ core–shell nanocubes/TiO_2_, which had a hydrogen production rate of 1.71  ±  0.33 µmol h^−1^, and 1.6 times higher than the Pd nanocubes/TiO_2_, with a rate of 1.46  ±  0.22 µmol h^−1^. In addition, the Pd@Pt_0.4_Pd_0.15_Ir_0.15_Ru_0.15_Rh_0.15‐4L_ core–shell nanocubes/TiO_2_ also exhibited higher catalytic activity compared to its counterpart, the Pd@Pt_0.4_Pd_0.15_Ir_0.15_Ru_0.15_Rh_0.15‐4L_ core–shell octahedra/TiO_2_ (≈1.31 µmol h^−1^), despite having an identical composition, highlighting the influence of distinct surface atomic arrangements on catalytic performance. Furthermore, as shown in Figure 3c, the turnover frequency (TOF), defined as hydrogen production per second per individual surface metal atom, was calculated to compare the intrinsic specific activity of the photocatalysts. Again, the Pd@Pt_0.4_Pd_0.15_Ir_0.15_Ru_0.15_Rh_0.15‐4L_ core–shell nanocubes/TiO_2_ exhibited the highest specific activity among all the photocatalysts in this study, achieving a TOF of 90 ±  19.4 h^−1^. We conducted control experiments under UV light illumination (*λ* = 400 nm) and continuous wavelength visible light illumination (*λ* > 420 nm) for Pd@HEA/TiO_2_. As shown in Figure S7 (Supporting Information), no detectable H_2_ production was observed in the visible light case. This confirms that the enhanced hydrogen production performance in the Pd@HEA/TiO_2_ system is due to the synergistic interaction between Pd@HEA and photoexcited TiO_2_, rather than a plasmonic effect from Pd@HEA.

In our study, we have systematically evaluated the stability of both the powder form and the thin‐film form of the Pd@Pt_0.4_Pd_0.15_Ir_0.15_Ru_0.15_Rh_0.15‐4L_ core–shell nanocubes supported on TiO_2_ under simulated solar light irradiation for 20 h. The test was performed for 5 cycles, and each cycle was maintained for 4 h under light irradiation. TEM images were taken under light irradiation at 4 and 20 h. As shown in Figure S8 (Supporting Information), the morphology of the photocatalyst did not exhibit significant changes. However, we observed that the catalytic activity gradually declined, which could potentially be attributed to either the surface reconstruction of composition or gradual aggregation of the photocatalysts during prolonged testing under magnetic stirring conditions, as shown in Figure S9 (Supporting Information).^[^ ^36^, ^37^, ^38^ ^]^ To avoid any aggregation issues, we further mixed TiO_2_ paste with Pd@Pt_0.4_Pd_0.15_Ir_0.15_Ru_0.15_Rh_0.15‐4L_ core–shell nanocubes uniformly and fabricated a thin film on a glass substrate for the photocatalytic test.^[^ ^39^ ^]^ Interestingly, in contrast to the powder form of the photocatalysts, the thin film of Pd@Pt_0.4_Pd_0.15_Ir_0.15_Ru_0.15_Rh_0.15‐4L_ core–shell nanocubes/TiO_2_ exhibited a slight decrease in activity after the first two cycles but then maintained almost constant activity over the subsequent three cycles. This observation suggests that surface reconstruction of the Pd@Pt_0.4_Pd_0.15_Ir_0.15_Ru_0.15_Rh_0.15‐4L_ core–shell nanocubes may have influenced the initial stages of photocatalysis, but the structure eventually stabilized, resulting in consistent catalytic hydrogen production thereafter.^[^ ^36^, ^37^, ^38^ ^]^

### 2.3 Prolonged Carrier Lifetime Revealed by UV Photoelectron and Transient Absorption Spectroscopies

**Figure 4:**
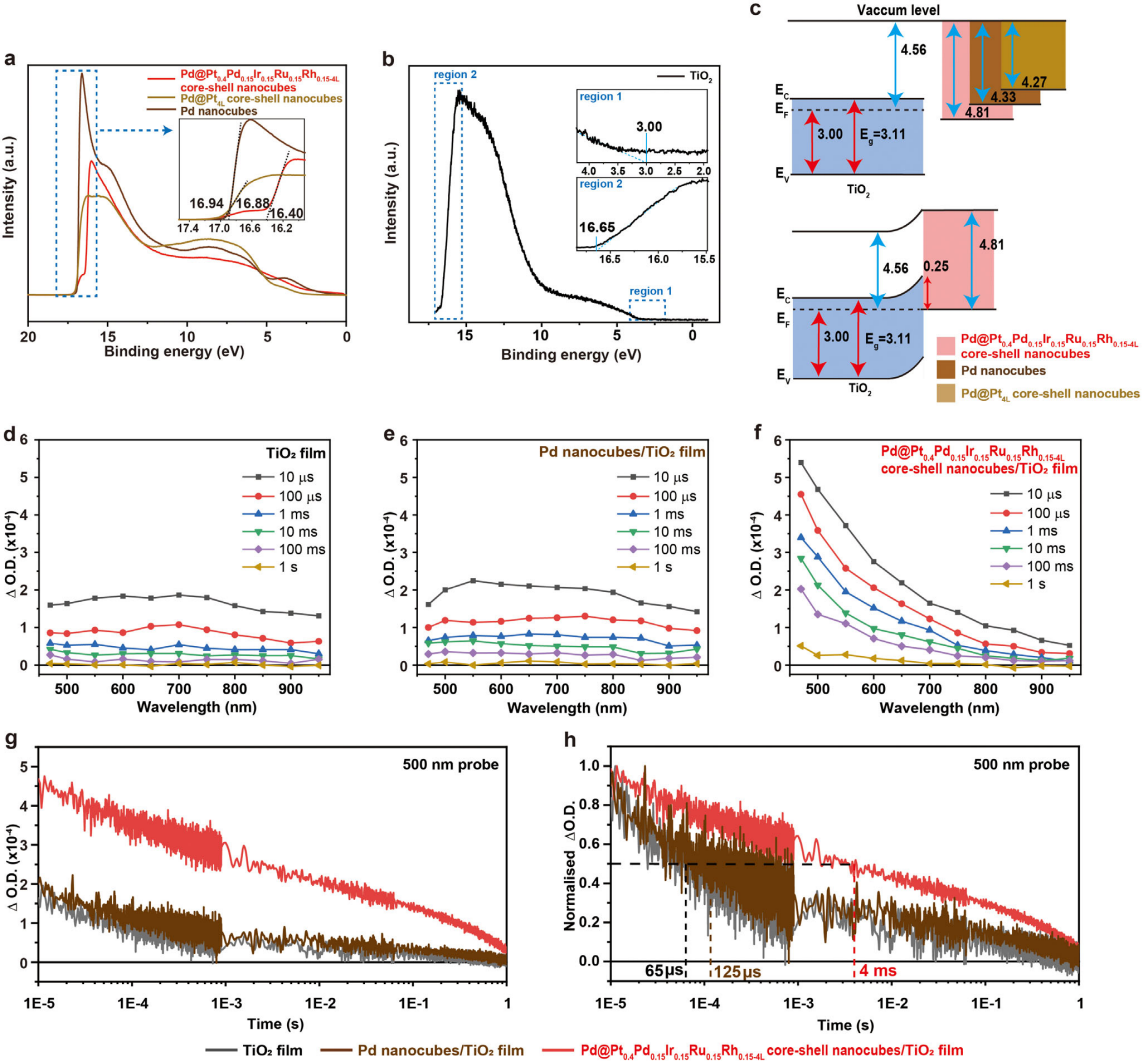
Band diagrams and carrier lifetime analysis. UPS spectra of a) Pd@Pt_0.4_Pd_0.15_Ir_0.15_Ru_0.15_Rh_0.15‐4L_ core–shell nanocubes, Pd@Pt_4L_ core–shell nanocubes and Pd nanocubes, and b) Pd@Pt_0.4_Pd_0.15_Ir_0.15_Ru_0.15_Rh_0.15‐4L_ core–shell nanocubes/TiO_2_ (inserts are enlarged figures depicting the valence band edge (region 1) and cut‐off edge (region 2). c) Band diagrams of metal and TiO_2_ before and after contact. TAS of d) TiO_2_, e) Pd nanocubes/TiO_2_, and f) Pd@Pt_0.4_Pd_0.15_Ir_0.15_Ru_0.15_Rh_0.15‐4L_ core–shell nanocubes/TiO_2_ on microsecond to second timescale following excitation at 355 nm (400 µJ cm^−2^ per pulse) at selected time delays. g) The TA decay kinetics of holes and h) their normalized traces were measured at a probe wavelength of 500 nm. The dashed line in h) denotes the temporal point at which the amplitude of the optical density change (ΔO.D) decreases by half.

**Figure** **4**a,b presents the UPS spectra obtained using He(I) radiation for Pd@Pt_0.4_Pd_0.15_Ir_0.15_Ru_0.15_Rh_0.15‐4L_ core–shell nanocubes, Pd@Pt_4L_ core–shell nanocubes, and Pd nanocubes. The work function (*Φ*) for each sample was determined using the equation of *Φ* = ℎν − *E*_cut‐off_ (where ℎ𝜈 corresponds to the incident photon energy: 21.21 eV, and E_cut‐off_ is the onset of secondary electron emission, as shown by the cut‐off edge position in the inset of Figure 4a).^[^ ^40^, ^41^ ^]^ The calculated work functions for Pd@Pt_0.4_Pd_0.15_Ir_0.15_Ru_0.15_Rh_0.15‐4L_ core–shell nanocubes, Pd@Pt_4L_ core–shell nanocubes, and Pd nanocubes are 4.81, 4.27, and 4.33 eV, respectively. Figure 4b displays the UPS spectra for pure TiO_2_. The work function and the valence band edge (*E*_v_) were identified as 4.56 and 3 eV, respectively, as shown in the inset of Figure 4b. Additionally, the band gap of TiO_2_ was calculated to be 3.11 eV using a Tauc plot derived from UV–vis spectroscopy (Figure S10, Supporting Information). Figure 4c illustrates the energy band diagrams for the nanocrystals/TiO_2_ interface before and after contact. Upon reaching thermal equilibrium, the Fermi levels (*E*_f_) of the nanocrystals and TiO_2_ align, leading to charge redistribution across the interface. The difference in their work functions generates an interfacial dipole, which in turn modifies the band structure through band bending. For Pd@Pt_0.4_Pd_0.15_Ir_0.15_Ru_0.15_Rh_0.15‐4L_ core–shell nanocubes, the higher work function compared to the Fermi level of TiO_2_ results in upward band bending, leading to the formation of a Schottky barrier with a height of 0.25 eV at the interface (Figure 4c). This junction facilitates charge separation by directing holes toward the surface and electrons into the bulk of the material, effectively extending carrier lifetimes. Moreover, the Schottky barrier prevents back electron transfer into TiO_2_, reducing charge recombination. At the same time, the moderate barrier height ensures efficient electron transfer from TiO_2_ to the Pd@Pt_0.4_Pd_0.15_Ir_0.15_Ru_0.15_Rh_0.15‐4L_ core–shell nanocubes, enabling effective catalytic activity.^[^ ^42^, ^43^, ^44^ ^]^ The UPS spectra of Pd@Pt_0.2_Pd_0.2_Ir_0.2_Ru_0.2_Rh_0.2‐4L_ core–shell nanocubes were measured for comparison, and the calculated work function of ≈4.25 eV, as shown in Figure S11 (Supporting Information). This work function, being smaller than that of pure TiO_2_, prevents the formation of a Schottky barrier. While the photocatalytic performance shows a correlation with the work function, it is not the only factor governing charge separation and the suppression of recombination.^[^ ^44^ ^]^

To investigate the charge transfer dynamics between TiO_2_ and HEA core–shell nanocubes and the photocatalytic hydrogen production reaction mechanism, TAS studies were carried out. The commercial TiO_2_, Pd nanocubes/TiO_2,_ and Pd@Pt_0.4_Pd_0.15_Ir_0.15_Ru_0.15_Rh_0.15‐4L_ core–shell nanocubes/TiO_2_ coated glass films were prepared for TAS measurements to increase the signal‐to‐noise ratio and are written in the Experimental Section. Ultrafast TAS on ps‐ns timescale was used to monitor the initial processes following the UV excitation at 355 nm for TiO_2_ films, Pd nanocubes/TiO_2_ films_,_ and Pd@Pt_0.4_Pd_0.15_Ir_0.15_Ru_0.15_Rh_0.15‐4L_ core–shell nanocubes/TiO_2_ films (Figure S12, Supporting Information). Photogenerated charge carriers (electrons and holes) exhibit distinct spectral signatures in the UV/Vis region.^[^ ^45^, ^46^, ^47^, ^48^ ^]^ Generally, photoinduced absorption from 500 to 700 nm which sharply rises at shorter wavelengths, is attributed to photogenerated holes (Figure S13a, Supporting Information), while the broad photoinduced absorption >850 nm is assigned to TiO_2_ shallow trapped photoelectrons (Figure S13b,c, Supporting Information); however, it is important to recognize that these two spectral signatures have significant overlap. With the introduction of Pd or HEA nanocubes, a broad bleaching of Pd surface plasmon resonance can be observed within picosecond to nanosecond timescale, which overlaps with the TiO_2_ photohole absorption (Figures S14–S16, Supporting Information),^[^ ^49^ ^]^ further complicating the direct analysis of photohole kinetics. As in Figure S13b,c (Supporting Information), the ultrafast TAS kinetics at 850 nm indicates that the addition of Pd nanocubes or Pd@Pt_0.4_Pd_0.15_Ir_0.15_Ru_0.15_Rh_0.15‐4L_ core–shell nanocubes shows no clear difference in the lifetime of TiO_2_ trapped photoelectrons on the ps‐ns timescale. To further probe the photogenerated charge carriers’ lifetimes, TAS measurements on the microsecond to the second timescale were performed (Figure 4d–h). The lifetime of TiO_2_ photoholes shows a very slight increase with the introduction of Pd nanocubes, while the deposition of Pd@Pt_0.4_Pd_0.15_Ir_0.15_Ru_0.15_Rh_0.15‐4L_ core–shell nanocubes onto TiO_2_ results in more than twofold‐increase TAS amplitude of TiO_2_ photoholes, and the lifetime of these holes is increased by ≈61 times (*t*_50%_, defined as the time required for a 50% decrease in the amplitude of photoinduced absorption at 10 µs, with TiO_2_ at ≈65 µs, Pd nanocubes/TiO_2_ at ≈125 µs and Pd@Pt_0.4_Pd_0.15_Ir_0.15_Ru_0.15_Rh_0.15‐4L_ core–shell nanocubes/TiO_2_ at ≈4 ms). Table S4 (Supporting Information) reviews the lifetime of metal‐TiO_2_ in previous reports. It is also notable that the TAS signal assigned to shallow trapped electrons (850 nm) is significantly decreased in amplitude on the microsecond timescales with the Pd@Pt_0.4_Pd_0.15_Ir_0.15_Ru_0.15_Rh_0.15‐4L_ sample. These results suggest that the Pd@Pt_0.4_Pd_0.15_Ir_0.15_Ru_0.15_Rh_0.15‐4L_ core–shell nanocubes are able to effectively accept electrons. Critically when compared to the Pd nanocubes/TiO_2_ sample the longer lifetime of the holes in Pd@Pt_0.4_Pd_0.15_Ir_0.15_Ru_0.15_Rh_0.15‐4L_ demonstrates that recombination is significantly decreased, enabling photogenerated electrons to drive the photocatalytic hydrogen production reaction and the holes to participate in AA oxidation.

### 2.4 Pt‐Enriched Active Sites on the High‐Entropy‐Alloy Surfaces Identified by In Situ X‐Ray Photoelectron Spectroscopy and Density Functional Theory

**Figure 5:**
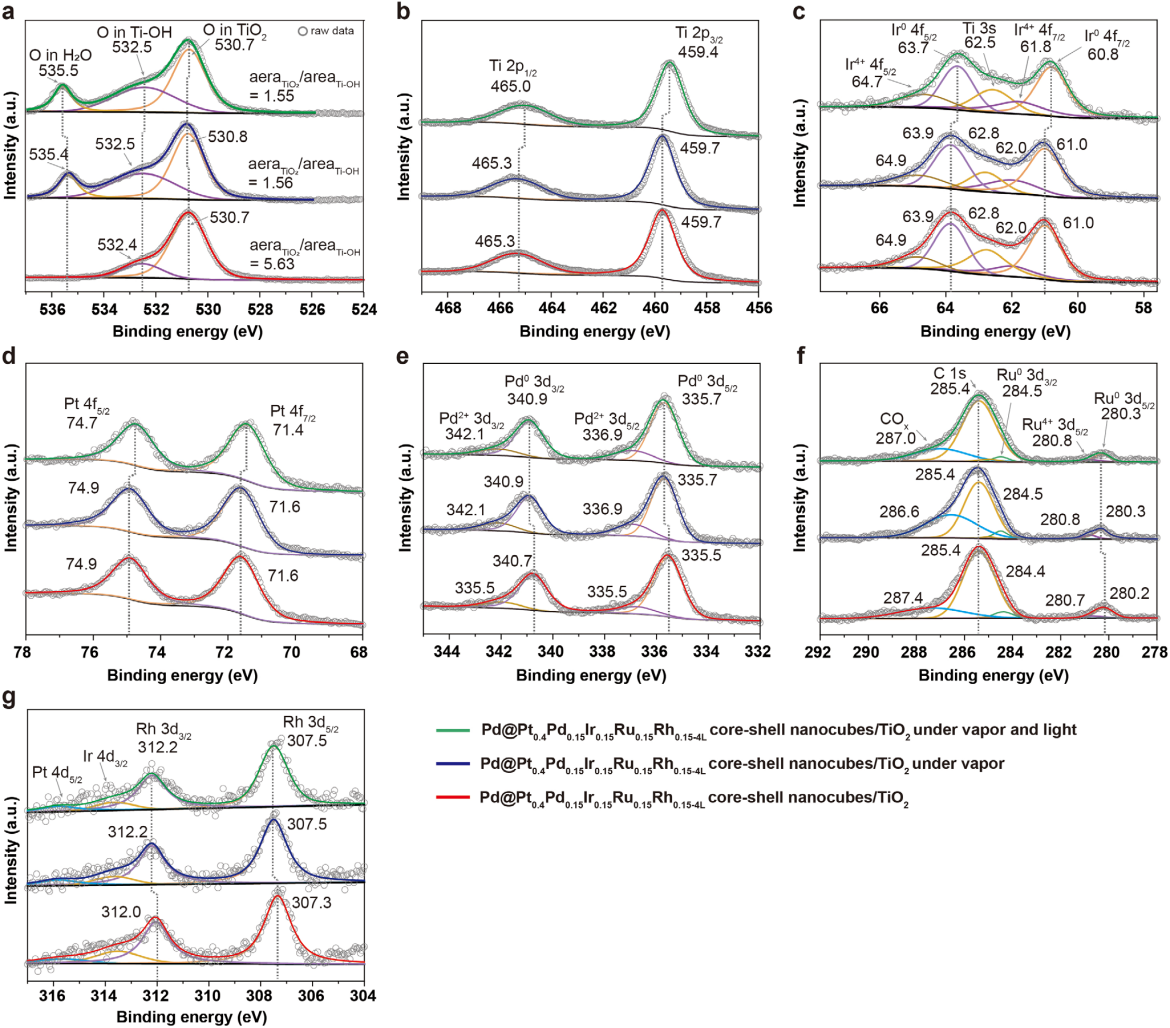
In situ synchrotron XPS analysis for Pd@Pt_0.4_Pd_0.15_Ir_0.15_Ru_0.15_Rh_0.15‐4L_ core–shell nanocubes/TiO_2_ during the photocatalytic hydrogen production. In situ XPS spectra of a) O 1s, b) Ti 2p, c) Ir 4f, d) Pt 4f, e) Pd 3d, f) Ru 3d and C 1s, and g) Pt 4d, Ir 4d, and Rh 3d under three different conditions: ultra‐high vacuum (red line), 0.3 mbar water vapor (blue line), and 0.3 mbar water vapor with simulated sunlight irradiation (green line; *λ *> 300 nm).

The underlying catalytic mechanisms and synergistic effects that underpin the exceptional performance of HEA catalysts remain poorly understood, primarily due to the limited exploration of these materials in photocatalysis.^[^ ^50^ ^]^ Advancing this field requires a deeper understanding of the synergistic interactions occurring on high‐entropy, multielement surfaces, as well as the identification of active catalytic sites. Employing in situ characterization techniques alongside theoretical calculations provides a powerful approach to unraveling these complexities. Such insights facilitate the rational design of HEA‐semiconductor photocatalysts, leveraging atomic‐scale synergies to enhance photocatalytic efficiency. We employed in situ synchrotron XPS analysis (**Figure** **5**, Table S5, Supporting Information) under three different conditions: ultra‐high vacuum (red line), 0.3 mbar water vapor (blue line), and 0.3 mbar water vapor with simulated sunlight irradiation (green line) to investigate the catalytic mechanism and also identify the adsorption sites of Pd@Pt_0.4_Pd_0.15_Ir_0.15_Ru_0.15_Rh_0.15‐4L_ core–shell nanocubes/TiO_2_ during photocatalytic hydrogen production. As shown in Figure 5a, under ultra‐high vacuum conditions, the asymmetrical O 1s peaks observed in the 538–524 eV range can be deconvoluted into contributions from lattice oxygen (Ti─O─Ti) within TiO_2_ and hydroxyl groups (Ti─OH) present on the TiO_2_ surface.^[^ ^51^ ^]^ When exposed to 0.3 mbar of water vapor, both without and with simulated sunlight irradiation, an additional peak centered ≈535.5 eV appears, corresponding to the oxygen from water vapor (H_2_O). For the Ti 2p peaks, exposure to 0.3 mbar of water vapor under simulated sunlight irradiation leads to a decrease in binding energy, likely caused by the rapid transfer of holes to the sacrificial agents, as shown in Figure 5b. This process increases the electron density in the TiO_2_, resulting in the observed shift in binding energy. Interestingly, the in situ XPS spectra of pure TiO_2_ (without the deposition of Pd@Pt_0.4_Pd_0.15_Ir_0.15_Ru_0.15_Rh_0.15‐4L_ core–shell nanocubes) reveal that the Ti 2p peaks remained unchanged throughout the photocatalytic process (Figure S17, Supporting Information). This observation confirms that the presence of core–shell nanocrystals is crucial for facilitating charge carrier separation, which subsequently initiates the water‐splitting reaction.^[^ ^52^ ^]^

By applying curve fitting, the Ir 4f_7/2_ and 4f_5/2_ peaks and the overlapping Ti 3s peaks were accurately distinguished, as shown in Figure 5c. The metallic Ir^0^ 4f_7/2_ and 4f_5/2_ peaks were consistently centered ≈61 and 63.9 eV under ultra‐high vacuum as well as under 0.3 mbar of water vapor. Notably, when the sample was exposed to 0.3 mbar of water vapor and subjected to simulated sunlight irradiation, both peaks shifted to lower binding energies, ≈60.8 and 63.7 eV, respectively. For Pt, a very noticeable shift in the metallic Pt^0^ 4f_7/2_ and 4f_5/2_ peaks was observed, as shown in Figure 5d. When the sample was exposed to 0.3 mbar of water vapor and subjected to simulated sunlight irradiation, the peaks shifted from 71.6 and 74.9 eV to 71.4 and 74.7 eV, respectively. However, curve fitting in the Pd, Ru, and Rh cases revealed that the peak positions and shapes remained almost unchanged, as shown in Figure 5e–g. These results highlight the pronounced effect of light on the electronic structure of Ir and Pt on the HEA surface, indicating that photoexcited electrons are predominantly transferred to Ir and Pt atoms, thereby increasing their electron density.^[^ ^33^ ^]^ This enhanced electron density accounts for the observed shift in binding energies toward lower values. The accumulation of electrons at the Ir and Pt sites suggests that these elements serve as the primary adsorption or reaction centers for hydrogen during photocatalysis. In contrast, the XPS spectra of Pd, Rh, and Ru show minimal binding energy shifts, implying their limited involvement in electron accumulation or surface adsorption processes.

**Figure 6:**
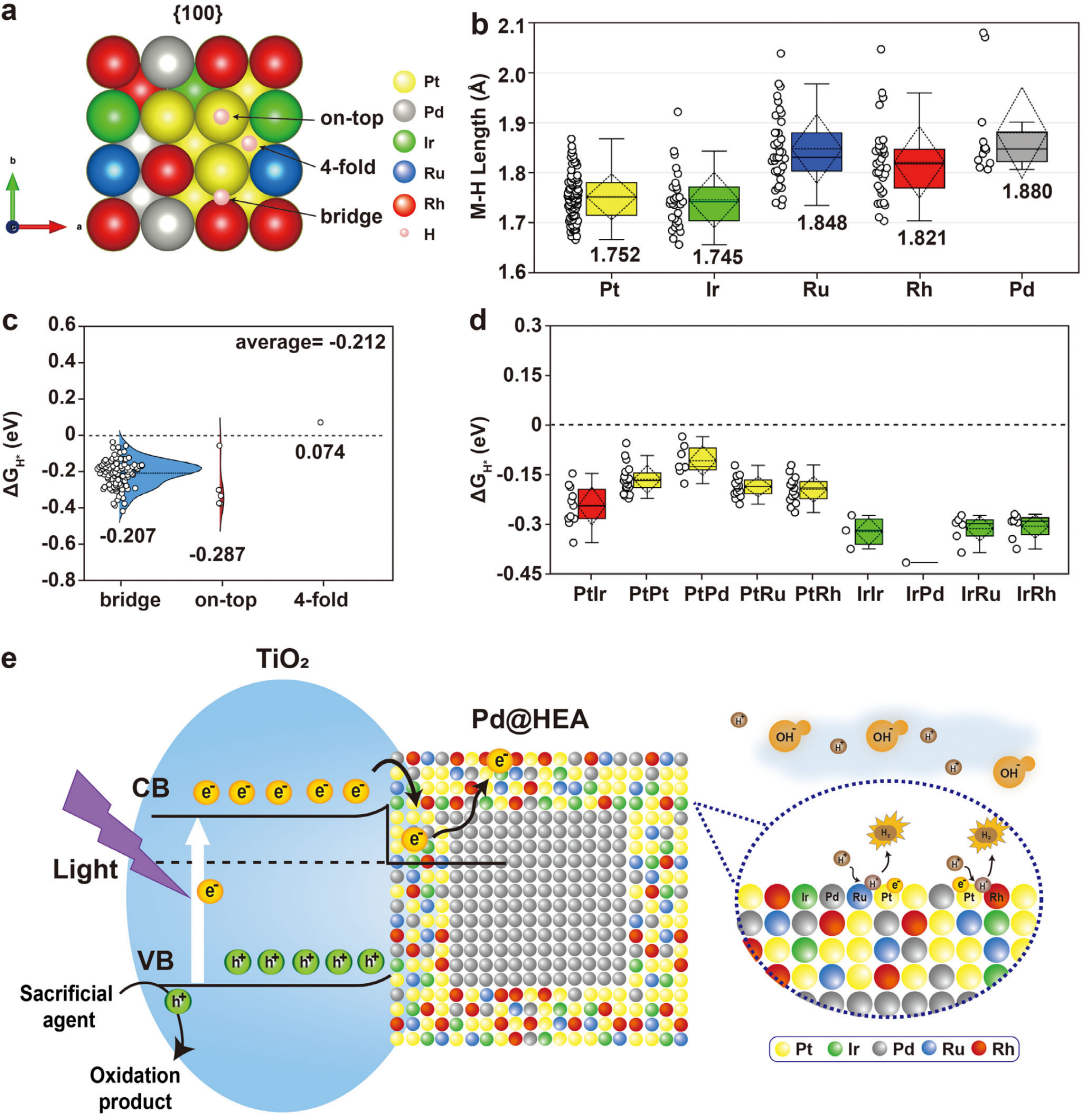
DFT calculations and schematic illustration of the photocatalytic hydrogen production mechanism for the Pd@Pt_0.4_Pd_0.15_Ir_0.15_Ru_0.15_Rh_0.15‐4L_ core–shell nanocubes/TiO_2_. a) Configurations of hydrogen adsorption on Pt_0.4_Pd_0.15_Ir_0.15_Ru_0.15_Rh_0.15_ {100} surfaces. b) Bond lengths of hydrogen adsorbed at bridge sites on different atomic species. c) Δ*G*_H*_ values and their distributions across the bridge, on‐top, and fourfold adsorption sites. d) Comparative analysis of Δ*G*_H*_ values at Pt‐based and Ir‐based bridge sites. e) Schematic mechanism of Pd@Pt_0.4_Pd_0.15_Ir_0.15_Ru_0.15_Rh_0.15‐4L_ core–shell nanocubes/TiO_2_ for photocatalytic hydrogen production.

To further elucidate the photocatalytic activity and active sites of Pd@Pt_0.4_Pd_0.15_Ir_0.15_Ru_0.15_Rh_0.15‐4L_ core–shell nanocubes, DFT calculations were performed, as shown in **Figure** **6**. The top and cross‐sectional views of the Pt_0.4_Pd_0.15_Ir_0.15_Ru_0.15_Rh_0.15_ {100} surface reveal a square atomic arrangement, where each atom is symmetrically coordinated with four nearest neighbors, forming on‐top, bridge, and fourfold adsorption sites, as shown in Figure 6a. In the DFT simulations, hydrogen atoms were initially placed at various potential adsorption sites on the constructed {100} atomic models. After structural optimization, the majority of hydrogen atoms migrated to bridge sites, indicating that these positions are the most energetically favorable for adsorption.^[^ ^17^ ^]^ We first analyzed the bond lengths of hydrogen adsorbed at bridge sites on different atomic species, as shown in Figure 6b. The results reveal that the average Pt─H (1.752 Å) and Ir─H (1.745 Å) bond lengths are shorter than those of Ru─H (1.848 Å), Rh─H (1.821 Å), and Pd─H (1.88 Å). These shorter bond distances indicate stronger hydrogen binding interactions at the Pt and Ir sites, identifying them as the primary adsorption centers. This conclusion is consistent with the in situ XPS results shown in Figure 5.

In photocatalytic hydrogen production, hydrogen serves as the primary reaction intermediate, and the hydrogen adsorption free energy (Δ*G*_H*_) is widely recognized as a key descriptor of catalytic performance.^[^ ^53^, ^54^ ^]^ Theoretical studies consistently demonstrate that when Δ*G*_H*_ approaches zero, the catalyst achieves an optimal hydrogen binding strength, thereby maximizing catalytic activity. This principle aligns with the Sabatier principle, which emphasizes that the interaction between a catalyst and its reaction intermediates must be optimally balanced, neither too strong nor too weak.^[^ ^55^ ^]^ As illustrated in Figure 6c, DFT calculations of Δ*G*_H*_ were conducted on over 100 possible atomic configurations for hydrogen‐adsorbed Pt_0.4_Pd_0.15_Ir_0.15_Ru_0.15_Rh_0.15_ {100} surfaces. The results revealed an average Δ*G*_H*_ value of −0.212 eV. Notably, the abundance of adsorption sites at bridge sites suggests that, upon structural relaxation, hydrogen preferentially adsorbs at bridge sites rather than on‐top or fourfold sites. Furthermore, the narrow Δ*G*_H*_ distribution at these bridge sites indicates more uniform and consistent adsorption behavior compared to on‐top sites. To determine whether Pt or Ir serves as the primary active site, a comparative analysis was conducted on the {100} surfaces for nine distinct compositional configurations: PtIr, PtPt, PtPd, PtRu, PtRh, IrIr, IrPd, IrRu, and IrRh (Figure 6d). The results show that Ir‐based bridge sites exhibit more negative Δ*G*_H*_ values, indicating stronger hydrogen adsorption. In contrast, Pt‐based bridge sites exhibit Δ*G*_H*_ values closer to zero, suggesting that Pt provides a more optimal adsorption strength. For instance, the IrRu bridge sites display a relatively low Δ*G*_H*_ value of −0.314 eV, whereas the corresponding PtRu bridge sites achieve a Δ*G*_H*_ value of −0.187 eV.^[^ ^56^, ^57^ ^]^ Furthermore, we evaluated the photocatalytic activities of Pd@Pt nanocubes/TiO_2_ core–shell nanocubes (Figure 3) and Pd@PtIr nanocubes/TiO_2_ core–shell nanocubes (Figure S18, Supporting Information). These catalysts exhibited lower hydrogen production rates of 1.65 and 0.8 µmol h^−1^, respectively, compared to Pd@Pt_0.4_Pd_0.15_Ir_0.15_Ru_0.15_Rh_0.15‐4L_ core–shell nanocubes/TiO_2_ (2.13 µmol h^−1^). This performance enhancement suggests that incorporating additional elements such as Pd, Ru, and Rh, despite their limited direct involvement in hydrogen adsorption, contributes to improved photocatalytic activity. This improvement is likely attributed to the “cocktail effect” characteristic of high‐entropy surfaces, where the synergistic interactions among multiple elements result in superior catalytic performance beyond what is achievable with conventional binary or ternary systems. Notably, the HEA surface in this work contains a high Pt content of 40 at.%, making it the most abundant element among the five constituents. This high surface concentration, combined with the favorable Δ*G*_H*_ of Pt‐based sites, strongly supports the conclusion that Pt serves as the dominant active site responsible for driving photocatalytic hydrogen production. Taken together, these findings demonstrate that the excellent active sites in HEAs are manifested through the high density of catalytically favorable Pt centers embedded within a cooperative multimetallic surface environment. Interestingly, while DFT calculations indicate that Pd@Pt_0.2_Pd_0.2_Ir_0.2_Ru_0.2_Rh_0.2_ {100} surfaces exhibit a Δ*G*_H*_ value (−0.198 eV; as shown in Figure S19, Supporting Information) slightly closer to the ideal range compared to Pt_0.4_Pd_0.15_Ir_0.15_Ru_0.15_Rh_0.15_ {100} surfaces (−0.212 eV), the latter demonstrates better photocatalytic activity (Figure 6c; Figure S20, Supporting Information). This discrepancy may be attributed to the higher Schottky barrier formation (Figure 4; Figure S11, Supporting Information) between Pd@Pt_0.4_Pd_0.15_Ir_0.15_Ru_0.15_Rh_0.15‐4L_ and TiO₂, along with the high surface concentration of Pt‐based sites, which collectively enhance charge separation and promote overall reaction efficiency.

The photocatalytic hydrogen production over Pd@Pt_0.4_Pd_0.15_Ir_0.15_Ru_0.15_Rh_0.15‐4L_ core–shell nanocubes/TiO_2_ in the presence of an AA as a sacrificial agent is depicted in Figure 6e. Upon illumination, photons excite electrons from the valence band to the conduction band of TiO_2_. These photoexcited electrons are subsequently transferred across the Schottky barrier to the Pd@Pt_0.4_Pd_0.15_Ir_0.15_Ru_0.15_Rh_0.15‐4L_ core–shell nanocubes. The HEA structure uniquely stabilizes these electrons, effectively prolonging their lifetime (Figure 4).^[^ ^42^ ^]^ Electron accumulation is primarily localized on Pt and Ir atoms at the HEA surface, which serve as the main hydrogen adsorption sites (Figure 5). Notably, Pt sites are more likely to act as the dominant active sites for hydrogen production, as the Δ*G*_H*_ values of Pt‐based bridge sites are closer to zero than those of Ir‐based sites (Figure 6), indicating a more optimal hydrogen binding strength. Simultaneously, AA acts as an efficient hole scavenger, capturing photogenerated holes and facilitating the oxidation half‐reaction.^[^ ^58^ ^]^ This synergy between effective charge separation and stabilization, enabled by the HEA and sacrificial agent, sustains robust photocatalytic performance and enhances electron availability for hydrogen production.

## 3 Conclusion

In this study, we successfully synthesized Pd@HEA core–shell nanocrystals with controlled compositions and facets on TiO_2_ supports, achieving significantly enhanced photocatalytic hydrogen production performance. Compared to Pd@Pt_4L_/TiO_2_ nanocubes, Pd@Pt_0.4_Pd_0.15_Ir_0.15_Ru_0.15_Rh_0.15‐4L_ core–shell nanocubes/TiO_2_ exhibited superior hydrogen production rates, which can be attributed to their optimized Schottky junctions and the synergistic interactions among multiple elements. UPS measurements revealed that Pd@Pt_0.4_Pd_0.15_Ir_0.15_Ru_0.15_Rh_0.15‐4L_ core–shell nanocubes exhibited a higher work function (4.81 eV) than Pd@Pt_4L_, facilitating improved charge separation. TAS further demonstrated that the carrier lifetime of Pd@Pt_0.4_Pd_0.15_Ir_0.15_Ru_0.15_Rh_0.15‐4L_ nanocubes/TiO_2_ extended to 4 ms, a significant increase compared to pure TiO_2_ (65 µs). In situ XPS analysis confirmed that Pt and Ir sites underwent notable binding energy shifts under light illumination, identifying them as primary hydrogen adsorption sites. DFT calculations further corroborated these findings, demonstrating that Pt‐based bridge sites exhibit an optimal hydrogen binding free energy (Δ*G*_H*_ = −0.187 eV), making Pt the dominant active site in photocatalytic reactions. These results highlight the crucial role of Pt‐enriched HEA surfaces in extending charge carrier lifetimes and enhancing photocatalytic efficiency. We believe this systematic study provides valuable insights into HEA nanocatalyst design and paves the way for developing next‐generation photocatalysts with unprecedented efficiency for solar energy conversion.

## 4 Experimental Section

### Preparation of Seeds: Pd Nanocubes

The Pd nanocubes with an edge length of 17.6 nm were prepared following established methods with slight adjustments to the original procedure.^[^ ^59^ ^]^ In a 20‐mL vial, 8 mL of an aqueous mixture containing 600 mg of KBr, 60 mg of AA, and 105 mg of PVP was heated to 80 °C under magnetic stirring for 10 min. Following this, 3 mL of an aqueous solution of Na_2_PdCl_4_ (57 mg) was introduced all at once using a pipette. The vial was subsequently sealed and kept at 80 °C with constant stirring for 3 h. The Pd nanocubes were harvested by centrifugation, cleaned three times with water, and ultimately redispersed in EG for subsequent applications.

### Preparation of Seeds: Pd Octahedra

Pd octahedra with an edge length of 17.2 nm were prepared following previously reported methods with slight modifications.^[^ ^59^ ^]^ Specifically, 0.3 mL of a 10.8‐nm Pd nanocubes aqueous suspension (1.8 mg mL^−1^) was mixed with 8 mL of an aqueous solution containing 100 µL of HCHO and 105 mg of PVP in a 20‐mL vial. The mixture was pre‐heated at 60 °C for 10 min before a single‐step addition of 3 mL Na_2_PdCl_4_ aqueous solution (37.7 mg). The reaction vial was sealed and kept at 60 °C under magnetic stirring for 3 h. The resulting Pd octahedra were isolated by centrifugation, washed three times with water, and finally suspended in EG for later use.

### Synthesis of Pd@PtPdIrRuRh Core–Shell Nanocrystals (0, 20, 40, 60, 80, and 100 at% Pt in the Shell) Using Epitaxial Growth

The following was employed to prepare Pd@Pd_0.2_Pt_0.2_Ir_0.2_Ru_0.2_Rh_0.2_ core–shell nanocrystals using a standard epitaxial growth method. A 20‐mL vial was charged with 0.40 mL of Pd cubic or octahedral seed suspension in EG, along with 20 mg of AA, 100 mg of PVP, 60 mg of KBr, and 3.1 mL of EG.^[^ ^15^, ^59^ ^]^ This mixture was heated to 110 °C under magnetic stirring for 20 min, after which the temperature was gradually increased to 195 °C over 20 min. Next, 14 mL of a precursor solution containing five metal precursors: K_2_PtCl_4_, Na_2_PdCl_4_, H_2_IrCl_6_·xH_2_O, RuCl_3_·xH_2_O, and RhCl₃·xH_2_O—each at an equal molar concentration (0.042 µmol mL^−1^) in EG, was titrated into the reaction mixture using a syringe pump at a controlled rate of 0.8 mL h^−1^. The thickness of the HEA shell was tuned to four atomic layers by adjusting the concentration of the metal precursors. Furthermore, the stoichiometry of the HEA shell on the Pd seeds was customizable by modifying the ratio of metal precursors in the synthesis, for synthesis of the different samples with controlled Pt ratio in the HEA shells. During precursor addition, the reaction temperature was held constant at 195 °C. The resulting Pd@PtPdIrRuRh core–shell nanocrystals were collected by centrifugation, washed once with acetone and twice with water, and finally dispersed in water for subsequent use.

### Photocatalytic Measurements for Hydrogen Production

To prepare metal–semiconductor hybrid structures for use as photocatalysts, Pd@Pt_0.2_Pd_0.2_Ir_0.2_Ru_0.2_Rh_0.2‐4L_ core–shell nanocubes or octahedra were dispersed onto commercial TiO_2_ P25 nanoparticles. Initially, the TiO_2_ P25 nanoparticles were thoroughly cleaned by washing them twice with a 1 m NaOH solution followed by DI water to remove any HCl contamination from their surfaces. Subsequently, 0.05 mg of Pd@PtPdIrRuRh core–shell nanocrystals were introduced into a 1 mL suspension of TiO_2_ P25 (1 mg mL^−1^ in DI water). The mixture was sonicated for 20 min to ensure even dispersion, after which the hybrid structures were collected via centrifugation.

The resulting product was re‐dispersed in 2 mL of ethanol and subjected to a multi‐step annealing process. This included heating at 90 °C for 1 h, followed by 190 °C for 2 h in air, and finally annealing at 190 °C for 1 h under a 10% H_2_/N_2_ forming gas atmosphere. This procedure ensured the formation of well‐adhered metal–semiconductor hybrid structures with optimized surface properties for photocatalytic applications. To evaluate the photocatalytic performance, 1.05 mg of the photocatalyst, specifically, the Pd@PtPdIrRuRh/TiO_2_ P25 hybrid structures were dispersed in 10 mL of deionized water. Next, 704 mg of AA was added as a sacrificial agent, and the pH of the suspension was adjusted to 4.0 using a KOH solution. The prepared sample was transferred into a 40 mL reactor, sonicated to ensure uniform dispersion, and purged with argon gas to remove any residual air. A 300 W Xenon lamp with a light intensity of 1000 W m^−2^ was used to irradiate the suspension during the photocatalytic reaction, which was carried out for 4 h. The hydrogen gas produced during the reaction was quantified using gas chromatography (GC).

The TOF of a catalyst was a key parameter that quantifies its efficiency, defined as the number of H_2_ generated per active site per second, under the assumption that the catalytic activity was restricted to the surface atoms of the metal nanocrystals. This approach simplified the analysis by focusing only on the atoms directly accessible for the hydrogen production reaction. Accordingly, the TOF could be determined using the following formula:^[^ ^60^, ^61^ ^]^

(1) $$\mathrm{TOF}\;=\frac{\mathrm{Number}\;\mathrm{of}\;\mathrm{hydrogen}\;\mathrm{production}\;\mathrm{per}\;\mathrm{second}\;}{\mathrm{Number}\;\mathrm{of}\;\mathrm{surface}\;\mathrm{atoms}\;}$$

The number of hydrogen production per second could be calculated by following the formula:

(2) $$\begin{array}{ccc} & & \mathrm{Average}\;\mathrm{per}\;\mathrm{second}\;H_{2}\;\mathrm{evolved}\;\left(\frac{\mathrm{mol}}{\mathrm{mg}\times s}\right)\times \mathrm{co}-\mathrm{catalyst}\;\mathrm{weight}\;\left(\mathrm{mg}\right)\;\\ & & \;\;\;\times \;\left(\frac{6.022\times 10^{23}}{mol}\right) \end{array}$$

The number of surface atoms on one cube and octahedral particle was ≈25 000 and 29 000, and the particle weight was ≈53 400 000 and 62 300 000 (g mol^−1^), respectively. The calculations were performed in the next section.

### Determination of the Ratio between Surface Atoms and the Total Number of Atoms in Core–Shell Nanocube

It was assumed that the ideal cubic geometry of the Pd nanocubes remained intact following the layer‐by‐layer epitaxial deposition of the desired metal layers. To estimate the structural composition, the total number of atoms in the core–shell nanocube and the number of atoms specifically within the shell comprising *n* layers were determined using the equations provided below:^[^ ^15^ ^]^

(3) $$\mathrm{The}\;\mathrm{total}\;\mathrm{number}\;\mathrm{of}\;\mathrm{atoms}=4\times \left(\frac{l^{3}}{A^{3}}\right)$$

(4) $$\begin{array}{ccc} & & \mathrm{The}\;\mathrm{total}\;\mathrm{number}\;\mathrm{of}\;\mathrm{atoms}\;\mathrm{in}\;\mathrm{the}\;\mathrm{shell}\;\left(n\;\mathrm{layers}\right)\\ & & =\sum_{L=1}^{n}\left\{\frac{6\;\times \;2\;\times \;{\left(\;l+a\;\times \;L\right)}^{2}}{a^{2}}\right\} \end{array}$$

where *l* is the edge length of Pd nanocubes (17.6 nm), *A* is the lattice constant of Pd (0.389 nm), and *a* is the lattice constant of the HEA layers (0.42 nm).

When the *n* is 4, the result of the calculation is:$\frac{\mathrm{The}\;\mathrm{total}\;\mathrm{number}\;\mathrm{of}\;\mathrm{atoms}\;\mathrm{in}\;\mathrm{the}\;\mathrm{shell}\;(4\;\mathrm{layers})\;}{\mathrm{The}\;\mathrm{total}\;\mathrm{number}\;\mathrm{of}\;\mathrm{atoms}}=20.36\;\mathrm{at}\%$.

### Determination of the Ratio between Surface Atoms and the Total Number of Atoms in Core–Shell Otcahedra

It was presumed that the truncated octahedral morphology of the Pd nanocrystals remained intact after the facet engineering process. This unique structure can be characterized using two parameters: *n_l_*, which represents the number of atoms along the edges of the complete octahedron, and *n_cut_*, which denotes the number of atomic layers removed to create the truncation compared to an ideal octahedron. Based on these parameters, the total number of atoms in the truncated core–shell octahedron and the number of atoms specifically in the shell comprising *n* layers were calculated using the following equations:^[^ ^62^ ^]^

(5) $$\mathrm{The}\;\mathrm{total}\;\mathrm{number}\;\mathrm{of}\;\mathrm{atoms}:\frac{1}{3}\left({2n}_{l}^{3}+n_{l}\right)\;-{2n}_{\mathrm{cut}}^{3}-{3n}_{\mathrm{cut}}^{2}-n_{\mathrm{cut}}$$

The total number of atoms in the shell (*n* layers):

(6) $$\begin{array}{ccc} & & \sum_{L=1}^{n}6{\left[\left(n_{\mathrm{cut}}+1\right)+2L\right]}^{2}\\ & & \;\;+8\left\{\frac{1}{2}\left[1+\left(n_{l}+2L\right)\right]\times \left(n_{l}+2L\right)-\frac{3}{2}\left[1+\left(n_{cut}+2L\right)\right]*\left(n_{cut}+2L\right)\right\}\\ & & \;\;-24\left[\left(n_{cut}+1\right)+2L]-12\;\times \;[\left(n_{l}-2n_{cut}\right)+2L\right] \end{array}$$

where *n_l_* and *n_cut_* are 83.33 (18.33 nm) and 33.45 (7.36 nm), respectively.

When the *n* is 4, the result of the calculation is:$\frac{\mathrm{The}\;\mathrm{total}\;\mathrm{number}\;\mathrm{of}\;\mathrm{atoms}\;\mathrm{in}\;\mathrm{the}\;\mathrm{shell}\;(4\;\mathrm{layers})\;}{\mathrm{The}\;\mathrm{total}\;\mathrm{number}\;\mathrm{of}\;\mathrm{atoms}}=20.94\;\mathrm{at}\%$.

The provided equations allow the authors to determine the ideal at% of the shells in the Pd@HEA core–shell nanocrystals with four atomic layers. The element's contents in the shell analyzed by ICP‐OES are shown in Table S2 (Supporting Information), which were a little different from the theoretical value.

Kinetics analysis of the simulated numbers of PGM precursor ions remaining in the reaction solution and instantaneous percentages of deposited PGM atoms: Since the reductant (AA) was in excess relative to the metal precursors, its concentration remained nearly constant during the reaction, allowing the rate law to be simplified to pseudo‐first‐order:^[^ ^63^ ^]^

(7) $$\ln\;{\left[M^{X+}\right]}_{t}=-\mathrm{kt}+\ln\;{\left[M^{X+}\right]}_{0}$$

where *k* is the combined rate constant (abbreviated as rate constant in the following text); [M^X +^]_*t*_ and [M^X +^]_0_ represent the concentrations of a metal precursor at a specific time point *t* and *t *= 0. Then, the [M^X +^]_*t*_ was collected by experiment. Specifically, the reduction reaction of the five metal precursors was carried out, and small amounts of the reaction solution were periodically extracted at fixed time intervals. Each extracted sample was immediately quenched in an ice bath and prepared for the following quantified test. Quantified using ICP‐OES, allows for the determination of *k* through curve fittings. Based on the previous study,^[^ ^15^, ^64^, ^65^ ^]^ the calculated values of *k* (s^−1^) are: Pd(II) = 2.18 × 10^−3^, Pt(II) = 3.19 × 10^−3^, Ir(IV) = 2.9 × 10^−3^, Ru(III) = 8.06 × 10^−4^, and Rh(III) = 3.25 × 10^−3^. Assuming a pseudo‐first‐order reaction and utilizing the *k* values for the PGM precursors, the depletion of metal precursor ions from each droplet could be calculated separately. The total amount of precursor ions remaining in the reaction mixture at time *t* (*n_t_*) is the sum of all droplets.^[^ ^63^ ^]^

(8) $$\begin{array}{ccc} & & n_{t}=n_{0}e^{-\mathrm{kt}}+n_{0}e^{-k\left(t-\tau \right)}+n_{0}e^{-k\left(t-2\tau \right)}+\cdots +n_{0}e^{-k\left(t-N\tau \right)}\\ & & =\frac{n_{0}e^{-\mathrm{kt}}\times \left(1-{\left(e^{k\tau }\right)}^{N+1}\right)}{1-e^{k\tau }} \end{array}$$

where *n_0_* is the number of precursor ions in a single droplet, *k* is the rate constant derived from the curve fitting of experimental data, τ represents the time gap between adjacent drops, and *N* denotes the total number of droplets introduced up to time *t*. With n_*t*_ derived above, further kinetic simulations could be conducted. First, the number of metal atoms formed from the reduction of precursor ions between successive droplets was calculated as (*n*_0_  −  (*n*_*t*_ −  *n*_*t*  −  τ_)), allowing the instantaneous percentages of the five metal atoms to be determined. Using the equation for mixing entropy, $\Delta S_{\mathrm{mix}}=-R\;\sum_{i=1}^{n}x_{i}\ln x_{i}$, the instantaneous entropy of mixing as a function of time could be estimated based on these data. Additionally, the total number of metal atoms generated, ((*n*_0_  ×  *N*)  −  *n_t_*), for each precursor up to time *t* could be used to calculate the temporal composition of shell atoms in the product.

### In Situ XPS Measurements

XPS measurements were conducted at the Taiwan Light Source (TLS) 24A1 beamline at NSRRC. The XPS system featured a focused spot size of 0.3 mm (*V*) × 0.7 mm (*H*). The nanocrystal coating on the ITO glass substrate was transferred into the ultra‐high vacuum XPS chamber (≈10^−8^ mbar) to minimize contamination. Spectra were acquired using 780 eV photon energy with a step of 0.05 eV, and each measurement was repeated 12 times to generate high‐resolution data by averaging and overlapping the spectra into a single plot. The binding energy scale was calibrated against the Au 4f_7/2_ peak at 84 eV using a separate Au foil as the reference standard. The experimental setup for in situ XPS measurements involved three distinct conditions. First, measurements were conducted under ultra‐high vacuum (≈10^−5 ^mbar) with low‐intensity LED ambient light to ensure no photocatalytic reactions occurred. Second, water vapor was gradually introduced by connecting the ultra‐high vacuum chamber to a water flask, carefully adjusting the valve to raise the pressure to 0.3 mbar. Finally, following the same condition, the chamber was irradiated with a high‐intensity Xe light source through a window, delivering sufficient energy to drive photocatalytic reactions.

XPS peak curve fitting was performed according to established rules: the area ratio of the 4f_7/2_ to 4f_5/2_ peaks_5_ was set to 4:3, the 3d_5/2_ to 3d_3/2_ ratio was set to 3:2, and the FWHM of separated peaks was constrained to remain identical. Additionally, the energy separation (*Δ*) between separated peaks for the same metal was ensured to be consistent across all conditions. By these guidelines, the analysis of peak shifts under different conditions provided precise insights into electron transfer dynamics.

### TAS Measurements

TAS in the fs‐ns timescale were recorded using a Harpia‐TAS (Light Conversion). The excitation light was produced by a Pharos‐SP‐10 W laser (Light Conversion, FWHM ≈140 fs, 10 kHz, 1030 nm) coupled with an optical parametric amplifier (Orpheus) to generate light at either 355 or 460 nm with a power of 750 µW (5 kHz). A white light probe was created by focusing the 1030 nm output onto a sapphire crystal within the Harpia spectrometer. The pump beam (≈0.6 mm in diameter) and the probe beam (≈0.4 mm in diameter) were aligned at the sample position. The data was processed using Carpetview software (Light Conversion).

The TAS measurements on the µs‐s timescale were performed using the third harmonic output at 355 nm from a Nd: YAG laser (Continuum, Surelite I‐10, 532 nm, 6 ns pulse width). The excitation power was set to 0.40 mJ cm^−2^ at a repetition rate of 0.66 Hz. The laser beam was directed to the sample via a liquid light guide. A 100 W tungsten lamp, coupled with a monochromator (OBB Corp., typically configured for a 4 nm resolution), served as the probe light source. The optical density variation (ΔO.D) of the sample was determined by measuring the transmitted light intensity, which was recorded by a silicon photodiode linked to a custom amplification system. This system was connected to both an oscilloscope (Tektronix TDS 220) for microsecond timescale data analysis and a data acquisition card (National Instruments NI‐6221) for millisecond‐to‐second data capture. To improve the signal‐to‐noise ratio, the data were averaged over 200 laser pulses for each wavelength.

The TiO_2_ film sample preparation method was shown in the previous works. Briefly, a doctor‐blade method was used to prepare the TiO_2_ film on glass. A commercial Titania paste (Aldrich) was selected as the TiO_2_. This paste was applied to form a thin film on a glass slide. Subsequently, the slide was placed in a Muffle furnace and subjected to a heat treatment at 490 °C for a duration of 2 h, and then in a 10% H_2_/N_2_ environment at 190 °C for 1 h, with the temperature being ramped up at a rate of 20 °C min^−1^. Once the heating process was complete, the slide was allowed to cool down to room temperature, resulting in a TiO_2_‐coated glass slide ready for TAS measurements.

The procedure for preparing the Pd nanocubes/TiO_2_ films and Pd@Pt_0.4_Pd_0.15_Ir_0.15_Ru_0.15_Rh_0.15‐4L_ core–shell nanocubes/TiO_2_ films glass slide involved sputtering the 1 mL methanol solution, containing Pd nanocubes or Pd@Pt_0.4_Pd_0.15_Ir_0.15_Ru_0.15_Rh_0.15‐4L_ core–shell nanocrystals at a concentration of 0.05 mg mL^−1^. After degassing the solution with Ar for 3 min, the mixture was exposed to a 365 nm LED light for 1 min, while continuously supplying argon gas. Subsequently, the slide underwent calcination at 190 °C for 2 h in a 10% H_2_/N_2_ environment, with the temperature being increased at a rate of 20 °C min^−1^.

### DFT Calculations

The catalytic behavior of FCC PtPdIrRuRh surfaces and their impact on hydrogen adsorption were investigated. First‐principles DFT calculations were performed using the Vienna Ab initio Simulation Package,^[^ ^66^ ^]^ employing the revised Perdew—Burke–Ernzerhof functional to account for exchange‐correlation interactions and the projector augmented wave method for accurate core‐electron treatment.^[^ ^67^ ^]^ To ensure an optimal balance between computational cost and precision, the plane‐wave basis set cutoff energy was set to 360 eV. The k‐point sampling was carried out using the Monkhorst–Pack scheme with a 7 × 7 × 1 grid for the {100} surface.^[^ ^68^ ^]^ Structural optimization was performed with a convergence criterion that limited residual atomic forces to below 0.02 eV Å^−1^ (EDIFFG = −0.02). Further computational details could be found in the “Computational details” section.

### Computational Details

To model the properties of Pd@Pt_0.4_Pd_0.15_Ir_0.15_Ru_0.15_Rh_0.15_ core–shell nanocubes, a 3 × 3 five‐layer {100} slab was constructed based on an FCC crystal structure with a lattice parameter of 3.91 Å. In this {100} slab, Pt atoms constituted 40% of the total metal composition to accurately represent the HEA surface of Pd@Pt_0.4_Pd_0.15_Ir_0.15_Ru_0.15_Rh_0.15_ nanocubes. Specifically, the slab contained 18 Pt atoms, along with 6 Ir, 7 Pd, 7 Ru, and 7 Rh atoms. For hydrogen adsorption calculations, the adsorption free energy of hydrogen (Δ*G*_H*_) serves as a critical descriptor for evaluating catalytic efficiency in photocatalytic hydrogen production. An optimal Δ*G*_H*_ value, ideally close to zero, signifies favorable reaction conditions, consistent with trends reported in prior research. The Δ*G*_H*_ is determined using the following equation:

(9) $$\begin{array}{ccc} \Delta G_{H*} & = & G\left(H\;\;*\right)-G\left(*\right)-1/2G\left(H_{2}\right)\approx E\left(H\;\;*\right)-E\left(*\right)-1/2E\left(H_{2}\right)\\ & & +\;\;0.24\left(\mathrm{in}\mathrm{eV}\right) \end{array}$$

In this equation, *E*(H*) and *E*(*) denote the electronic energies of the slab with an adsorbed hydrogen atom and the clean slab, respectively. A correction term of 0.24 eV accounts for zero‐point energy and entropy contributions. This approach allowed for a systematic evaluation of photocatalytic activity across various materials, offering critical insights for the development and refinement of high‐performance catalysts. During the geometry optimization process, some initial adsorption configurations converged to identical final structures. The total number of unique hydrogen adsorption sites identified for {100} surface is summarized in Figure S20 (Supporting Information).

## Conflict of Interest

The authors declare no conflict of interest.

## Supporting information



Supporting Information

## Data Availability

The data that support the findings of this study are available from the corresponding author upon reasonable request.

## References

[1] L. Yuan , Z. Geng , J. Xu , F. Guo , C. Han , Adv. Funct. Mater. 2021, 31, 2101103.

[2] A. Jian , W. Li , M. Wang , H. Jia , S. Sang , Micro Nano Lett. 2021, 16, 601.

[3] B. Mei , K. Han , G. Mul , ACS Catal. 2018, 8, 9154. 30319883 10.1021/acscatal.8b02215 PMC6179457

[4] M. Luo , P. Lu , W. Yao , C. Huang , Q. Xu , Q. Wu , Y. Kuwahara , H. Yamashita , ACS Appl. Mater. Interfaces 2016, 8, 20667. 27439590 10.1021/acsami.6b04388

[5] A. R. Poerwoprajitno , L. Gloag , S. Cheong , J. J. Gooding , R. D. Tilley , Nanoscale 2019, 11, 18995. 31403640 10.1039/c9nr05802h

[6] M. Shi , D. Luo , P. Wu , Y. Shen , J. Wei , S. Guo , Z. Lu , Y. Huang , Y. Ni , Sci. China Mater. 2024, 67, 824.

[7] K. H. Ye , H. Li , D. Huang , S. Xiao , W. Qiu , M. Li , Y. Hu , W. Mai , H. Ji , S. Yang , Nat. Commun. 2019, 10, 3687. 31417082 10.1038/s41467-019-11586-y PMC6695449

[8] Z. Chen , T. Ma , W. Wei , W. Y. Wong , C. Zhao , B. J. Ni , Adv. Mater. 2024, 36, 2401568. 10.1002/adma.202401568 38682861

[9] Y. Sun , S. Dai , Sci. Adv. 2021, 7, 1600. 10.1126/sciadv.abg1600 PMC8115918 33980494

[10] L. Sun , K. Wen , G. Li , X. Zhang , X. Zeng , B. Johannessen , S. Zhang , ACS Mater. Au 2024, 4, 547. 39554860 10.1021/acsmaterialsau.4c00080 PMC11565283

[11] J. W. Yeh , S. K. Chen , S. J. Lin , J. Y. Gan , T. S. Chin , T. T. Shun , C. H. Tsau , S. Y. Chang , Adv. Eng. Mater. 2004, 6, 299.

[12] Y. Yao , Q. Dong , A. Brozena , J. Luo , J. Miao , M. Chi , C. Wang , I. G. Kevrekidis , Z. J. Ren , J. Greeley , Science 2022, 376, abn3103. 10.1126/science.abn3103 35389801

[13] Z. W. Chen , J. Li , P. Ou , J. E. Huang , Z. Wen , L. Chen , X. Yao , G. Cai , C. C. Yang , C. V. Singh , Nat. Commun. 2024, 15, 359. 38191599 10.1038/s41467-023-44261-4 PMC10774414

[14] D. Wu , K. Kusada , T. Yamamoto , T. Toriyama , S. Matsumura , S. Kawaguchi , Y. Kubota , H. Kitagawa , J. Am. Chem. Soc. 2020, 142, 13833. 32786816 10.1021/jacs.0c04807

[15] Y. C. Hsiao , C. Y. Wu , C. H. Lee , W. Y. Huang , H. V. Thang , C. C. Chi , W. J. Zeng , J. Q. Gao , C. Y. Lin , J. T. Lin , A. M. Gardner , H. Jang , R. H. Juang , Y. H. Liu , I. M. A. Mekhemer , M. Y. Lu , Y. R. Lu , H. H. Chou , C. H. Kuo , S. Zhou , L. C. Hsu , H. Y. Tiffany Chen , A. J. Cowan , S. F. Hung , J. W. Yeh , T. H. Yang , Adv. Mater. 2025, 37, 2411464.

[16] T. H. Hu , C. Y. Wu , Z. Y. He , Y. Chen , L. C. Hsu , C. W. Pao , J. T. Lin , C. W. Chang , S. C. Lin , R. Osmundsen , L. Casalena , K. H. Lin , S. Zhou , T. H. Yang , Adv. Sci. 2025, 12, 2409023. 10.1002/advs.202409023 PMC11714166 39513371

[17] C. Y. Wu , Y. C. Hsiao , Y. Chen , K. H. Lin , T. J. Lee , C. C. Chi , J. T. Lin , L. C. Hsu , H. J. Tsai , J. Q. Gao , C. W. Chang , I. T. Kao , C. Y. Wu , Y. R. Lu , C. W. Pao , S. F. Hung , M. Y. Lu , S. Zhou , T. H. Yang , Sci. Adv. 2024, 10, adl3693. 10.1126/sciadv.adl3693 PMC11277269 39058768

[18] G. Feng , F. Ning , J. Song , H. Shang , K. Zhang , Z. Ding , P. Gao , W. Chu , D. Xia , J. Am. Chem. Soc. 2021, 143, 17117. 34554733 10.1021/jacs.1c07643

[19] J. Hao , Z. Zhuang , K. Cao , G. Gao , C. Wang , F. Lai , S. Lu , P. Ma , W. Dong , T. Liu , Nat. Commun. 2022, 13, 2662. 35562523 10.1038/s41467-022-30379-4 PMC9106752

[20] C. Zhan , Y. Xu , L. Bu , H. Zhu , Y. Feng , T. Yang , Y. Zhang , Z. Yang , B. Huang , Q. Shao , Nat. Commun. 2021, 12, 6261. 34716289 10.1038/s41467-021-26425-2 PMC8556242

[21] D. Wu , K. Kusada , Y. Nanba , M. Koyama , T. Yamamoto , T. Toriyama , S. Matsumura , O. Seo , I. Gueye , J. Kim , J. Am. Chem. Soc. 2022, 144, 3365. 35166532 10.1021/jacs.1c13616

[22] K. M. Urs , N. K. Katiyar , R. Kumar , K. Biswas , A. K. Singh , C. Tiwary , V. Kamble , Nanoscale 2020, 12, 11830. 32459255 10.1039/d0nr02177f

[23] S. Nie , L. Wu , Q. Zhang , Y. Huang , Q. Liu , X. Wang , Nat. Commun. 2024, 15, 6669. 39107324 10.1038/s41467-024-50977-8 PMC11303686

[24] H. Ling , H. Sun , L. Lu , J. Zhang , L. Liao , J. Wang , X. Zhang , Y. Lan , R. Li , W. Lu , L. Cai , X. Bai , W. Wang , Nat. Commun. 2024, 15, 9505. 39489764 10.1038/s41467-024-53896-w PMC11532407

[25] L. Sun , W. Wang , P. Lu , Q. Liu , L. Wang , H. Tang , Chin. J. Catal. 2023, 51, 90.

[26] X. Xia , S. Xie , M. Liu , H. C. Peng , N. Lu , J. Wang , M. J. Kim , Y. Xia , Proc. Natl. Acad. Sci. U. S. A. 2013, 110, 6669. 23569268 10.1073/pnas.1222109110 PMC3637688

[27] K. D. Gilroy , X. Yang , S. Xie , M. Zhao , D. Qin , Y. Xia , Adv. Mater. 2018, 30, 1706312. 10.1002/adma.201706312 29656471

[28] J. Liu , Y. Li , X. Zhou , H. Jiang , H. G. Yang , C. Li , J. Mater. Chem. A 2020, 8, 17.

[29] J. Park , L. Zhang , S.‐I. Choi , L. T. Roling , N. Lu , J. A. Herron , S. Xie , J. Wang , M. J. Kim , M. Mavrikakis , Y. Xia , ACS Nano 2015, 9, 2635. 25661922 10.1021/nn506387w

[30] J. Chastain , R. C. King Jr. , Perkin‐Elmer Minnesota 1992.

[31] J. H. Baek , J. Y. Park , J. S. Kang , D. Kim , S. W. Koh , Y. C. Kang , Bull. Korean Chem. Soc. 2012, 33, 2694.

[32] A. Thøgersen , J. Mayandi , L. Vines , M. F. Sunding , A. Olsen , S. Diplas , M. Mitome , Y. Bando , J. Appl. Phys. 2011, 109, 084329.

[33] M. K. Hossain , M. M. Hossain , S. Akhtar , ACS Omega 2023, 8, 1979. 36687086 10.1021/acsomega.2c05107 PMC9850748

[34] V. Kumaravel , M. D. Imam , A. Badreldin , R. K. Chava , J. Y. Do , M. Kang , A. Abdel‐Wahab , Catalysts 2019, 9, 276.

[35] S. Oros‐Ruiz , R. Zanella , R. López , A. Hernández‐Gordillo , R. Gómez , J. Hazard. Mater. 2013, 263, 2. 23608749 10.1016/j.jhazmat.2013.03.057

[36] M. I. Abdullah , Y. Fang , X. Wu , M. Hu , J. Shao , Y. Tao , H. Wang , Nat. Commun. 2024, 15, 10587. 39632899 10.1038/s41467-024-54987-4 PMC11618364

[37] F. A. Ospina Acevedo , J. F. Godínez Salomón , Z. G. Naymik , K. C. Matthews , J. H. Warner , C. P. Rhodes , P. B. Balbuena , J. Phys. Chem. C 2025, 129, 3595. 10.1021/acs.jpcc.4c08119 PMC11848923 40008199

[38] B. He , P. Hosseini , D. Escalera‐López , J. Schulwitz , O. Rüdiger , U. Hagemann , M. Heidelmann , S. DeBeer , M. Muhler , S. Cherevko , Adv. Energy Mater. 2024, 15, 2403096.

[39] J. Huo , J. P. Tessonnier , B. H. Shanks , ACS Catal. 2021, 11, 5248.

[40] T. H. Yang , L. D. Huang , Y. W. Harn , C. C. Lin , J. K. Chang , C. I. Wu , J. M. Wu , Small 2013, 9, 3169. 23650082 10.1002/smll.201300424

[41] H. Chen , S. Chen , X. Quan , H. Yu , H. Zhao , Y. Zhang , J. Phys. Chem. C 2008, 112, 9285.

[42] M. S. Arshad , Š. Trafela , K. Ž. Rožman , J. Kovač , P. Djinović , A. Pintar , J. Mater. Chem. C 2017, 5, 10509.

[43] J. Freitag , D. W. Bahnemann , ChemPhysChem 2015, 16, 2670. 26118550 10.1002/cphc.201500281

[44] C. Beasley , M. Kumaran Gnanamani , E. Santillan‐Jimenez , M. Martinelli , W. D. Shafer , S. D. Hopps , N. Wanninayake , D. Y. Kim , ChemistrySelect 2020, 5, 1013.

[45] X. Li , C. Li , Y. Xu , Q. Liu , M. Bahri , L. Zhang , N. D. Browning , A. J. Cowan , J. Tang , Nat. Energy 2023, 8, 1013.

[46] Y. Tamaki , A. Furube , M. Murai , K. Hara , R. Katoh , M. Tachiya , Phys. Chem. Chem. Phys. 2007, 9, 1453. 17356752 10.1039/b617552j

[47] D. W. Bahnemann , M. Hilgendorff , R. Memming , J. Phys. Chem. B 1997, 101, 4265.

[48] T. Yoshihara , R. Katoh , A. Furube , Y. Tamaki , M. Murai , K. Hara , S. Murata , H. Arakawa , M. Tachiya , J. Phys. Chem. B 2004, 108, 3817.

[49] R. Long , K. Mao , M. Gong , S. Zhou , J. Hu , M. Zhi , Y. You , S. Bai , J. Jiang , Q. Zhang , Angew. Chem., Int. Ed. 2014, 53, 3205. 10.1002/anie.201309660 24520003

[50] Y. Xin , S. Li , Y. Qian , W. Zhu , H. Yuan , P. Jiang , R. Guo , L. Wang , ACS Catal. 2020, 10, 11280.

[51] S. Benkoula , O. Sublemontier , M. Patanen , C. Nicolas , F. Sirotti , A. Naitabdi , F. Gaie‐Levrel , E. Antonsson , D. Aureau , F. X. Ouf , S. I. Wada , A. Etcheberry , K. Ueda , C. Miron , Sci. Rep. 2015, 5, 15088. 26462615 10.1038/srep15088 PMC4604456

[52] L. Wang , B. Cheng , L. Zhang , J. Yu , Small 2021, 17, 2103447. 10.1002/smll.202103447 34510752

[53] W. K. Chong , B. J. Ng , C. C. Er , L. L. Tan , S. P. Chai , Sci. Rep. 2022, 12, 1927. 35121781 10.1038/s41598-022-05740-8 PMC8817050

[54] J. Shen , C. Luo , S. Qiao , Y. Chen , K. Fu , J. Xu , J. Pei , Y. Tang , X. Zhang , H. Tang , Adv. Funct. Mater. 2024, 34, 2309056.

[55] A. B. Laursen , A. S. Varela , F. Dionigi , H. Fanchiu , C. Miller , O. L. Trinhammer , J. Rossmeisl , S. Dahl , J. Chem. Educ. 2012, 89, 1595.

[56] F. Li , G. F. Han , H. J. Noh , J. P. Jeon , I. Ahmad , S. Chen , C. Yang , Y. Bu , Z. Fu , Y. Lu , J. B. Baek , Nat. Commun. 2019, 10, 4060. 31492875 10.1038/s41467-019-12012-z PMC6731251

[57] C. Li , J. B. Baek , ACS Omega 2020, 5, 31. 32803082 10.1021/acsomega.0c02838 PMC7424710

[58] J. Zhang , J. Wang , Y. Tang , K. Liu , B. Zhang , G. Ma , ACS Appl. Mater. Interfaces 2022, 14, 34656. 35860844 10.1021/acsami.2c06302

[59] B. Lim , M. Jiang , J. Tao , P. H. Camargo , Y. Zhu , Y. Xia , Adv. Funct. Mater. 2009, 19, 189.

[60] J. N. Tiwari , S. Sultan , C. W. Myung , T. Yoon , N. Li , M. Ha , A. M. Harzandi , H. J. Park , D. Y. Kim , S. S. Chandrasekaran , W. G. Lee , V. Vij , H. Kang , T. J. Shin , H. S. Shin , G. Lee , Z. Lee , K. S. Kim , Nat. Energy 2018, 3, 773.

[61] S. Anantharaj , P. E. Karthik , S. Noda , Angew. Chem., Int. Ed. 2021, 60, 23051. 10.1002/anie.202110352 PMC8596788 34523770

[62] F. Baletto , R. Ferrando , Rev. Mod. Phys. 2005, 77, 371.

[63] H. C. Peng , J. Park , L. Zhang , Y. Xia , J. Am. Chem. Soc. 2015, 137, 6643. 25941798 10.1021/jacs.5b03040

[64] Y. H. Liu , C. J. Hsieh , L. C. Hsu , K. H. Lin , Y. C. Hsiao , C. C. Chi , J. T. Lin , C. W. Chang , S. C. Lin , C. Y. Wu , J. Q. Gao , C. W. Pao , Y. M. Chang , M. Y. Lu , S. Zhou , T. H. Yang , Sci. Adv. 2023, 9, adf9931. 10.1126/sciadv.adf9931 PMC10171813 37163597

[65] M. Zhou , H. Wang , M. Vara , Z. D. Hood , M. Luo , T.‐H. Yang , S. Bao , M. Chi , P. Xiao , Y. Zhang , Y. Xia , J. Am. Chem. Soc. 2016, 138, 12263. 27568848 10.1021/jacs.6b07213

[66] G. Kresse , J. Furthmüller , Phys. Rev. B 1996, 54, 11169. 10.1103/physrevb.54.11169 9984901

[67] B. Hammer , L. B. Hansen , J. K. Nørskov , Phys. Rev. B 1999, 59, 7413.

[68] G. Kresse , D. Joubert , Phys. Rev. B 1999, 59, 1758.

