# Supplementary material for: Spectroscopic and Theoretical Insights Into High‐Entropy‐Alloy Surfaces and Their Interfaces with Semiconductors for Enhanced Photocatalytic Hydrogen Production
Source: Small. 2025 May 8;21(25):2503512. doi: 10.1002/smll.202503512 (PMC12199105; doi:10.1002/smll.202503512)
Supplement: Supplementary file 1 — Supporting Information [file SMLL-21-2503512-s001.docx]

Supporting Information

**Spectroscopic and Theoretical Insights into High-Entropy-Alloy Surfaces and Their Interfaces with Semiconductors for Enhanced Photocatalytic Hydrogen Production**

Jui-Tai Lin,^1^ Yueh-Chun Hsiao,^1,2^ Chao Li,^2,3^ Ching-Yuan Tseng,^1^ Zong-Ying He,^4^ Adrian M. Gardner,^2,5^ Yi Chen,^1^ Chueh-Cheng Yang,^6^ Chia-Hsin Wang,^6^ Shang-Cheng Lin,^1^ Xin-Xuan Lin,^1^ Chih-Yi Lin,^1^ Kun-Han Lin,^1^ Alexander J. Cowan,^2^* and Tung-Han Yang^1,4,7^*

^1^Department of Chemical Engineering, National Tsing Hua University, Hsinchu 300044, Taiwan.

^2^Stephenson Institute for Renewable Energy and Department of Chemistry, University of Liverpool, Liverpool L69 7ZF, United Kingdom.

^3^Department of Chemistry, The Hong Kong University of Science and Technology, Kowloon, Hong Kong (SAR) 999077, China.

^4^College of Semiconductor Research, National Tsing Hua University, Hsinchu 300044, Taiwan.

^5^Early Career Laser Laboratory, University of Liverpool, Liverpool, L69 3BX, United Kingdom.

^6^National Synchrotron Radiation Research Center, Hsinchu 300092, Taiwan.

^7^High Entropy Materials Center, National Tsing Hua University, Hsinchu 300044, Taiwan.

*Corresponding author. Email: [tunghanyang@mx.nthu.edu.tw](mailto:tunghanyang@mx.nthu.edu.tw); [A.J.Cowan@liverpool.ac.uk](mailto:A.J.Cowan@liverpool.ac.uk)

This Supporting Information file includes:

Experimental Section, Figures S1 to S20, Tables S1 to S5, and References

**Experimental Section**

*Materials:* Sodium tetrachloropalladate (Na_2_PdCl_4_), potassium tetrachloroplatinate (K2PtCl_4_), dihydrogen hexachloroiridate (H_2_IrCl_6_·xH_2_O), rhodium chloride hydrate (RhCl_3_·xH_2_O), ruthenium chloride hydrate (RuCl_3_·xH_2_O), L-ascorbic acid (AA), poly(vinyl pyrrolidone) (PVP, MW ≈ 55,000), formaldehyde (HCHO), 37% hydrochloric acid (HCl), 98% sulfuric acid (H2SO_4_), potassium hydroxide (KOH), 2-propanol ((CH_3_)_2_CHOH), titanium dioxide (TiO_2_) P25, 19.0 wt% transparent titanium paste (TiO_2_), 5 wt% Nafion solution, and potassium bromide (KBr) were sourced from Sigma-Aldrich. Ethylene glycol (EG), 99.8% acetone (CH_3_COCH_3_), and 99.5% ethanol (C_2_H_5_OH) were procured from J. T. Baker. For all experiments, deionized (DI) water with a resistivity of 18.2 MΩ cm was utilized.

*Characterizations:* Elemental composition was determined using inductively coupled plasma optical emission spectroscopy (ICP-OES) on a Thermo SCIENTIFIC iCAP 7200 Duo instrument. Transmission electron microscopy (TEM), high-angle annular dark-field scanning electron microscopy (HAADF-STEM), and energy-dispersive spectroscopy (EDS) mapping were conducted using a spherical-aberration corrected field emission TEM (JEOL JEM-ARM200FTH), which was operated at 200 kV. The crystal structures were analyzed by X-ray diffraction (XRD) using a Bruker D8A25 diffractometer. Surface chemical analysis was performed with X-ray photoelectron spectroscopy (XPS) using a high-resolution X-ray photoelectron spectrometer (ULVAC-PHI, PHI Quantera II). The ultraviolet photoelectron spectroscopy (UPS) was measured by electron spectroscopy (ULVAC-PHI PHI 5000 Versaprobe II) with an ultraviolet source (He I= 21.21 eV). In situ XPS measurements of samples with external simulated sunlight irradiation are conducted at the Taiwan Light Source (TLS) 24A1 of the NSRRC. We used a GC (Shimadzu, Nexis GC-2030) to measure the amount of H_2_ production during photocatalytic hydrogen production. The ultraviolet–visible spectroscopy (UV) was measured by electron spectroscopy (Jasco V-730).


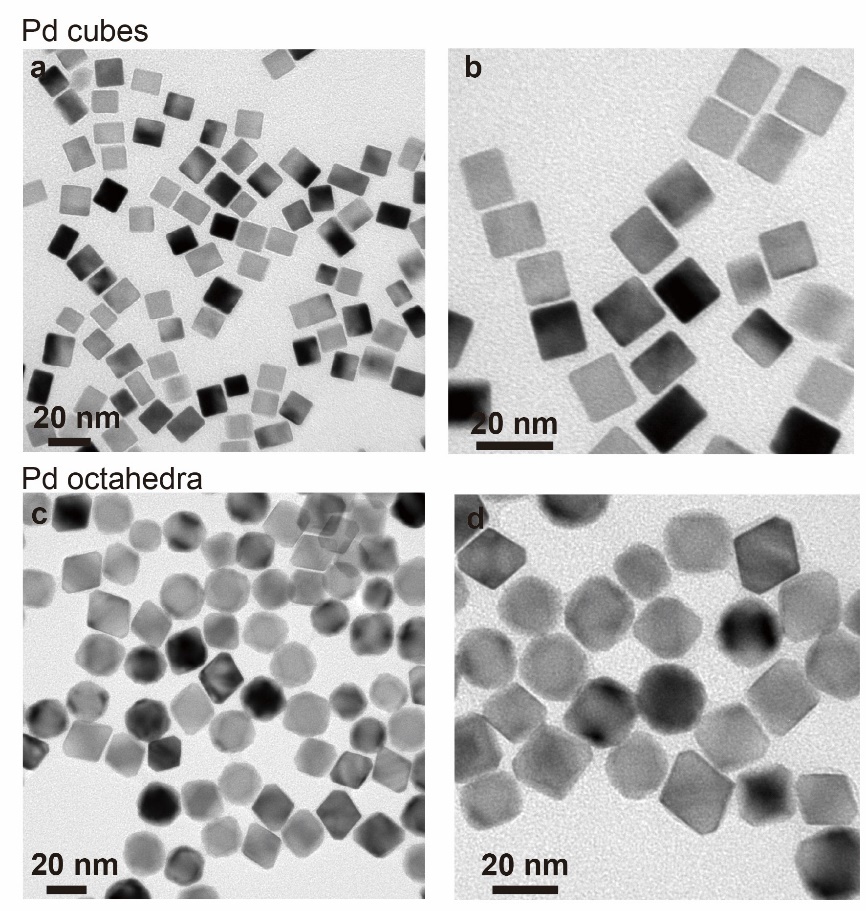


**Figure S1.** TEM images of a, b) 17.6-nm Pd nanocubes and c, d) 18.3-nm Pd octahedra as the seeds for the epitaxial growth of HEA atomic layers.


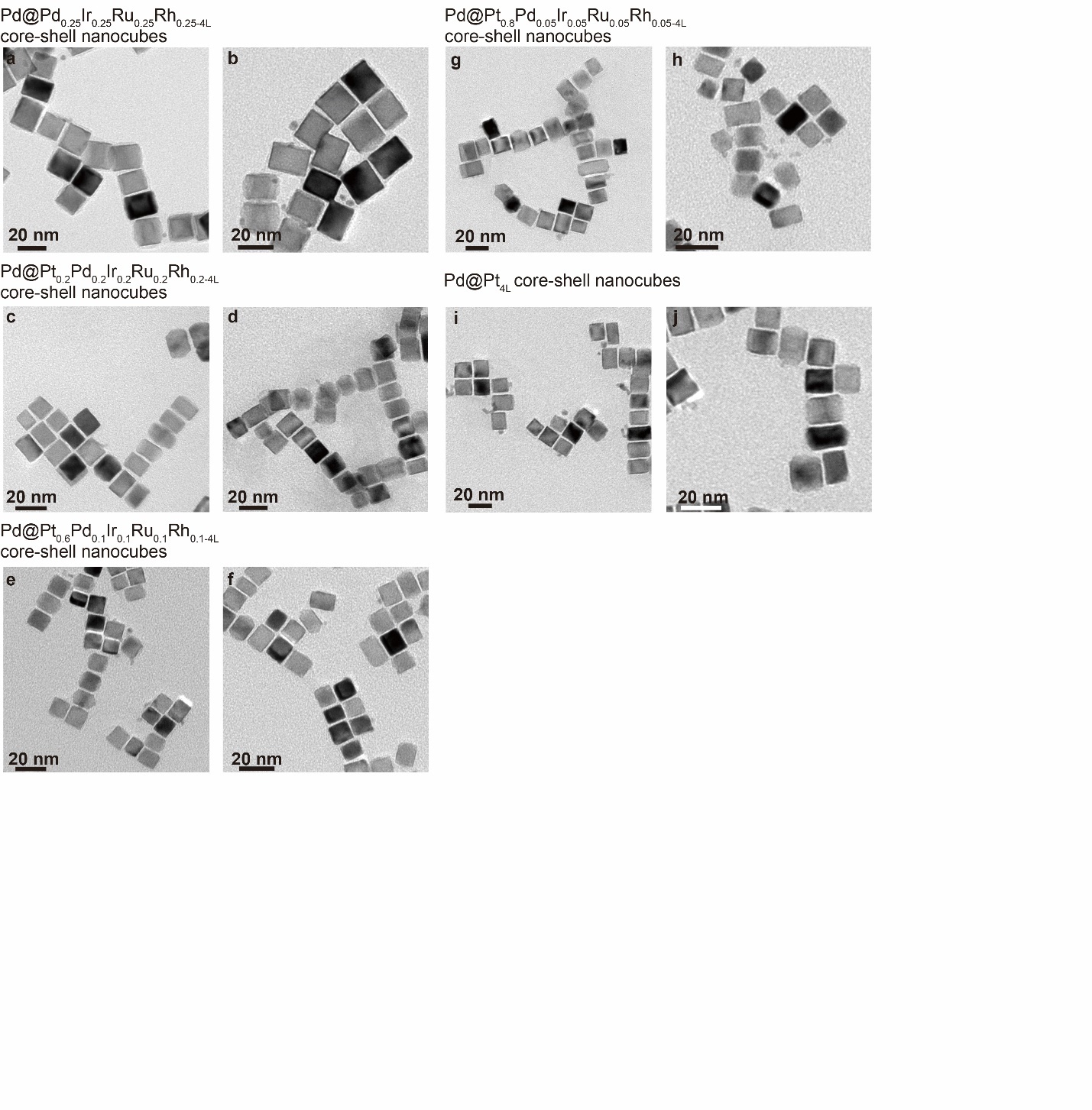


**Figure S2.** TEM images of a, b) Pd@Pd_0.25_Ir_0.25_Ru_0.25_Rh_0.25-4L_ core-shell nanocubes, c, d) Pd@Pt_0.2_Pd_0.2_Ir_0.2_Ru_0.2_Rh_0.2-4L_ core-shell nanocubes, e, f) Pd@Pt_0.6_Pd_0.1_Ir_0.1_Ru_0.1_Rh_0.1-4L_ core-shell nanocubes, g, h) Pd@Pt_0.8_Pd_0.05_Ir_0.05_Ru_0.05_Rh_0.05-4L_ core-shell nanocubes, and i, j) Pd@Pt_4L_ core-shell nanocubes.


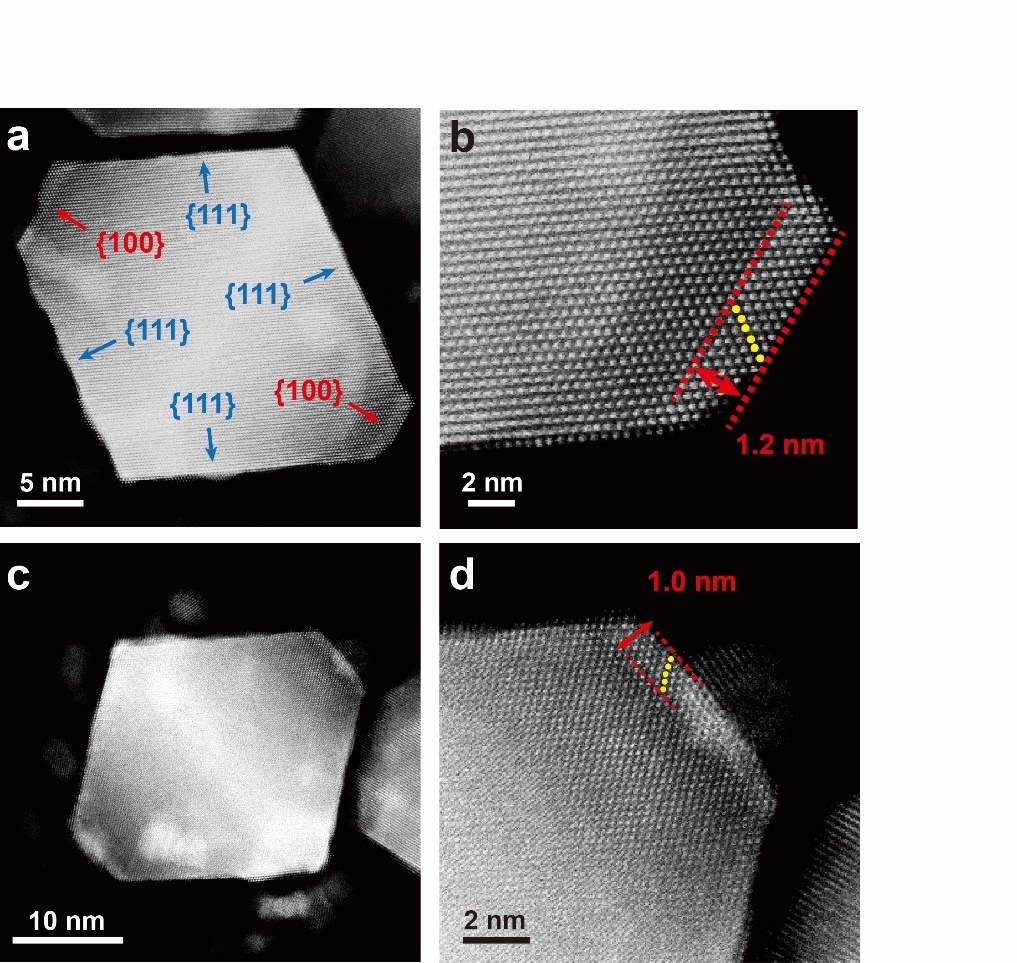


**Figure S3.** Atomic-resolution HAADF-STEM images for a, c) Pd@Pt_0.4_Pd_0.15_Ir_0.15_Ru_0.15_Rh_0.15-4L_ core-shell octahedron. b, d) The deposition of HEA atomic layers occurred preferentially on the {100} facets rather than the {111} surfaces, as thicker HEA atomic layers were observed on the {100} facets.

**
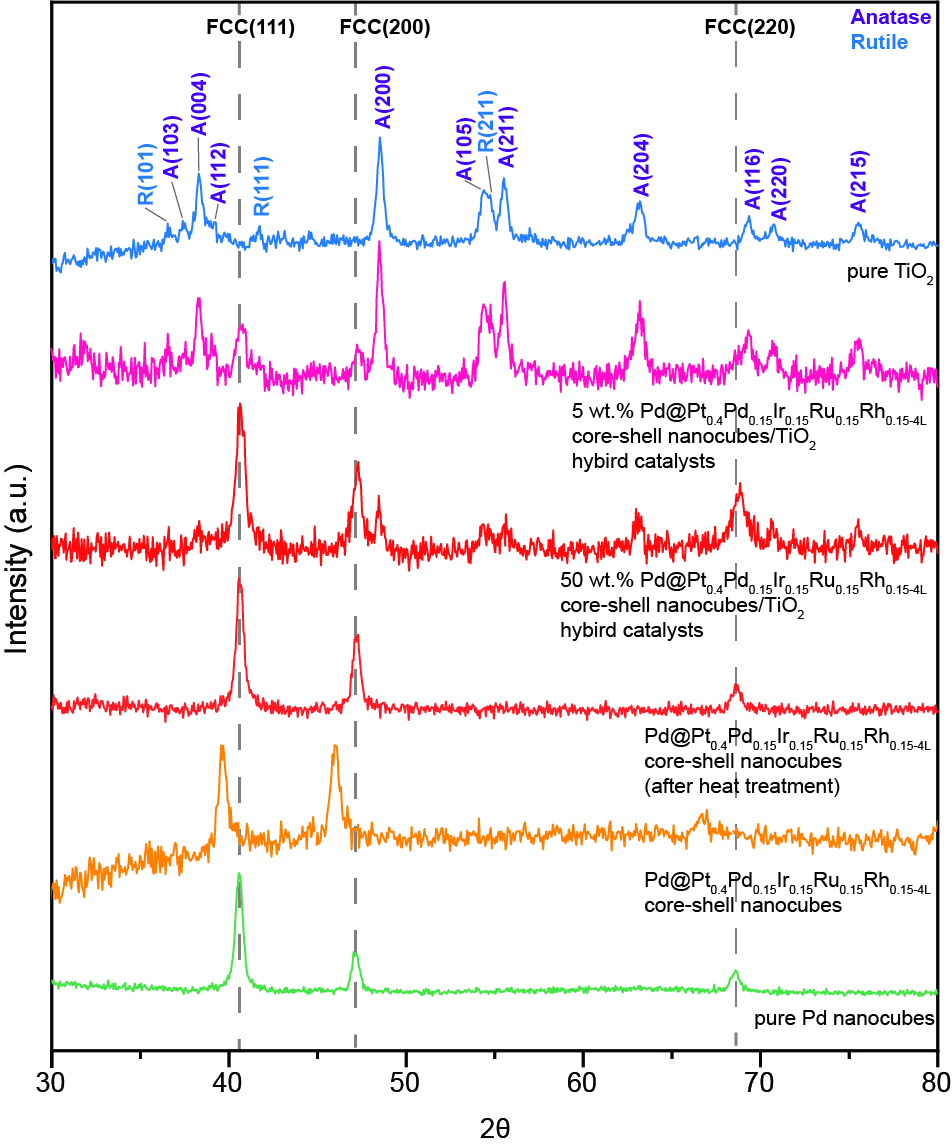
**

**Figure S4.** XRD analysis of the pure Pd nanocubes, Pd@Pt_0.4_Pd_0.15_Ir_0.15_Ru_0.15_Rh_0.15-4L_ core-shell nanocubes, Pd@Pt_0.4_Pd_0.15_Ir_0.15_Ru_0.15_Rh_0.15-4L_ core-shell nanocubes (after heat treatment), 50 wt.% Pd@Pt_0.4_Pd_0.15_Ir_0.15_Ru_0.15_Rh_0.15-4L_/TiO_2_ hybrid catalysts, 5 wt.% Pd@Pt_0.4_Pd_0.15_Ir_0.15_Ru_0.15_Rh_0.15-4L_/TiO_2_ hybrid catalysts, and pure TiO_2_. Notably, the 50 wt.% hybrid sample was specifically prepared to enhance the signal intensity of the FCC phase and provide clearer structural confirmation in the XRD pattern. For both the pure Pd nanocubes and the Pd@Pt_0.4_Pd_0.15_Ir_0.15_Ru_0.15_Rh_0.15-4L_ core-shell nanocubes, the XRD patterns exhibit three distinct peaks corresponding to the (111), (200), and (220) planes of a FCC structure, with no evidence of impurity phases or phase segregation. Upon deposition of the HEA shell, a slight shift of the diffraction peaks to lower angles was observed. This shift aligns well with our HRTEM and FFT results (**Figure 1**), further confirming the formation of uniform FCC-phase HEA shells on the Pd nanocube cores. After heat treatment, the FCC diffraction peaks of Pd@Pt_0.4_Pd_0.15_Ir_0.15_Ru_0.15_Rh_0.15-4L_ core-shell nanocubes (after heat treatment) shift back to higher angles, indicating lattice contraction due to annealing. Moreover, after dispersing the Pd@Pt_0.4_Pd_0.15_Ir_0.15_Ru_0.15_Rh_0.15-4L_ core-shell nanocubes onto TiO_2_ supports (comprising anatase and rutile phases) and performing thermal treatment, the FCC phase of Pd@Pt_0.4_Pd_0.15_Ir_0.15_Ru_0.15_Rh_0.15-4L_ core-shell nanocubes remained unchanged, indicating the structural stability of the HEA shells.


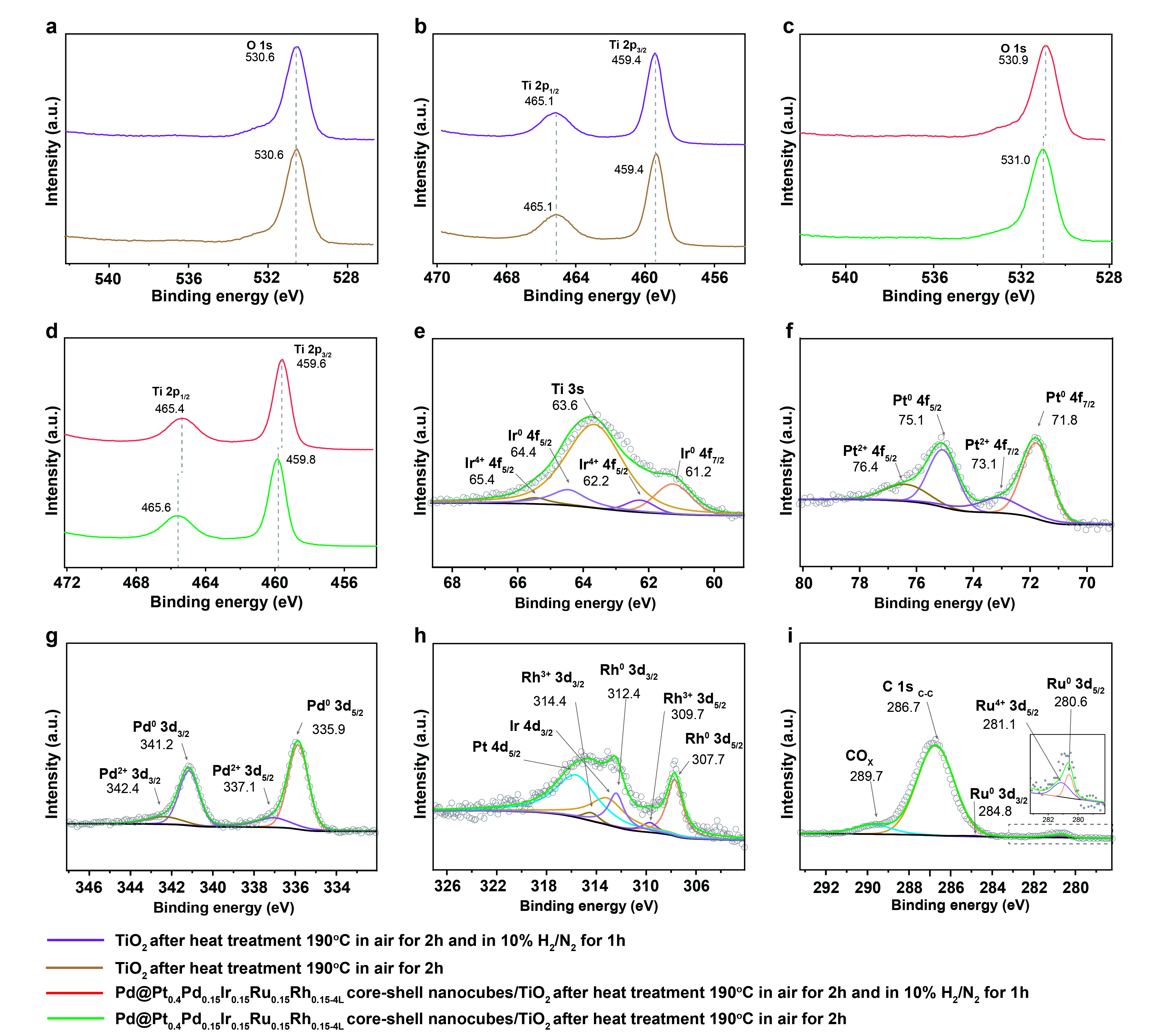


**Figure S5.** XPS spectra of the Pd@Pt_0.4_Pd_0.15_Ir_0.15_Ru_0.15_Rh_0.15-4L_ core-shell nanocubes/TiO_2_ and the TiO_2_ supports after different heat treatment steps. a, c) O 1s, b, d) Ti 2p, e) Ti 3s, Ir 4f, f) Pt 4f, g) Pd 3d, h) Pt 4d, Ir 4d, Rh 3d, i) C 1s and Ru 3d. The corresponding peak positions are summarized in **Table S3**. Notably, the Ti 3s binding energy in pure TiO_2_ remains unchanged across all heat treatment stages, indicating structural stability. In contrast, the binding energies of Pt, Pd, Ir, Ru, and Rh in Pd@HEA/ TiO_2_ shift to more reduced states after heat treatment in air, reflecting electron transfer between the Pd@HEA nanocrystals and the TiO_2_ support as well as the removal of surface organic residues. Subsequent heat treatment in a 10% H_2_/N_2_ atmosphere further eliminates surface oxides, yielding a cleaner metallic state, which enhances the electronic interaction between the HEA nanocubes and TiO_2_.

**
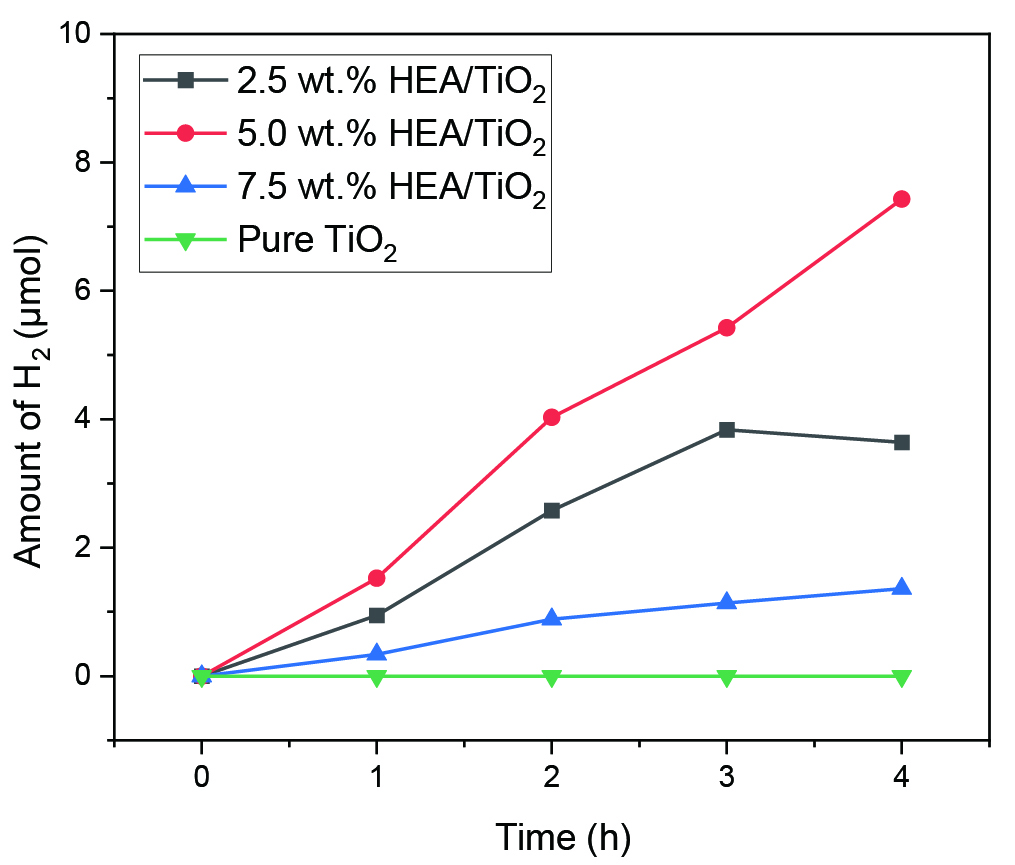
**

**Figure S6.** Photocatalytic hydrogen production. Control experiments were conducted using varying loading amounts of Pd@Pt_0.2_Pd_0.2_Ir_0.2_Ru_0.2_Rh_0.2-4L_ supported on TiO_2_ (HEA/TiO_2_) and pure TiO_2_ nanoparticles under simulated sunlight irradiation (λ > 300 nm) for 4 hours. No detectable hydrogen production was observed when using pure TiO_2_ nanoparticles, as determined by GC measurements.


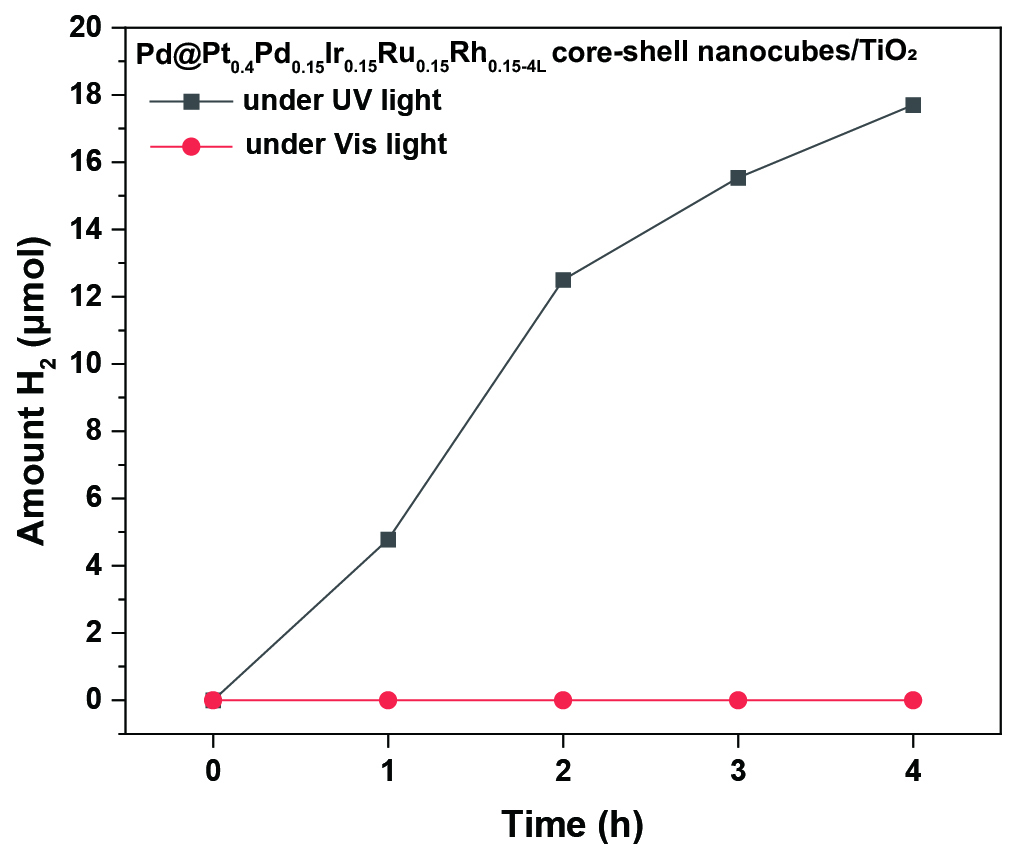


**Figure S7.** Photocatalytic hydrogen production. We conducted control experiments on Pd@Pt_0.4_Pd_0.15_Ir_0.15_Ru_0.15_Rh_0.15-4L_ core-shell nanocubes/TiO_2_ under UV light (λ = 400 nm) and visible light (λ > 420 nm) illumination. No detectable H_2_ production was observed under visible light, confirming that the enhanced hydrogen production in the Pd@HEA/TiO_2_ system is not due to the plasmonic effect of Pd@HEA. The UV light source was a single-wavelength LED (λ = 400 nm), capable of exciting TiO_2_, with a power density of 50 W m^-2^. The visible light source provided continuous wavelengths (λ > 420 nm), which do not excite TiO_2_, with a power density of 1000 W m^-2^.


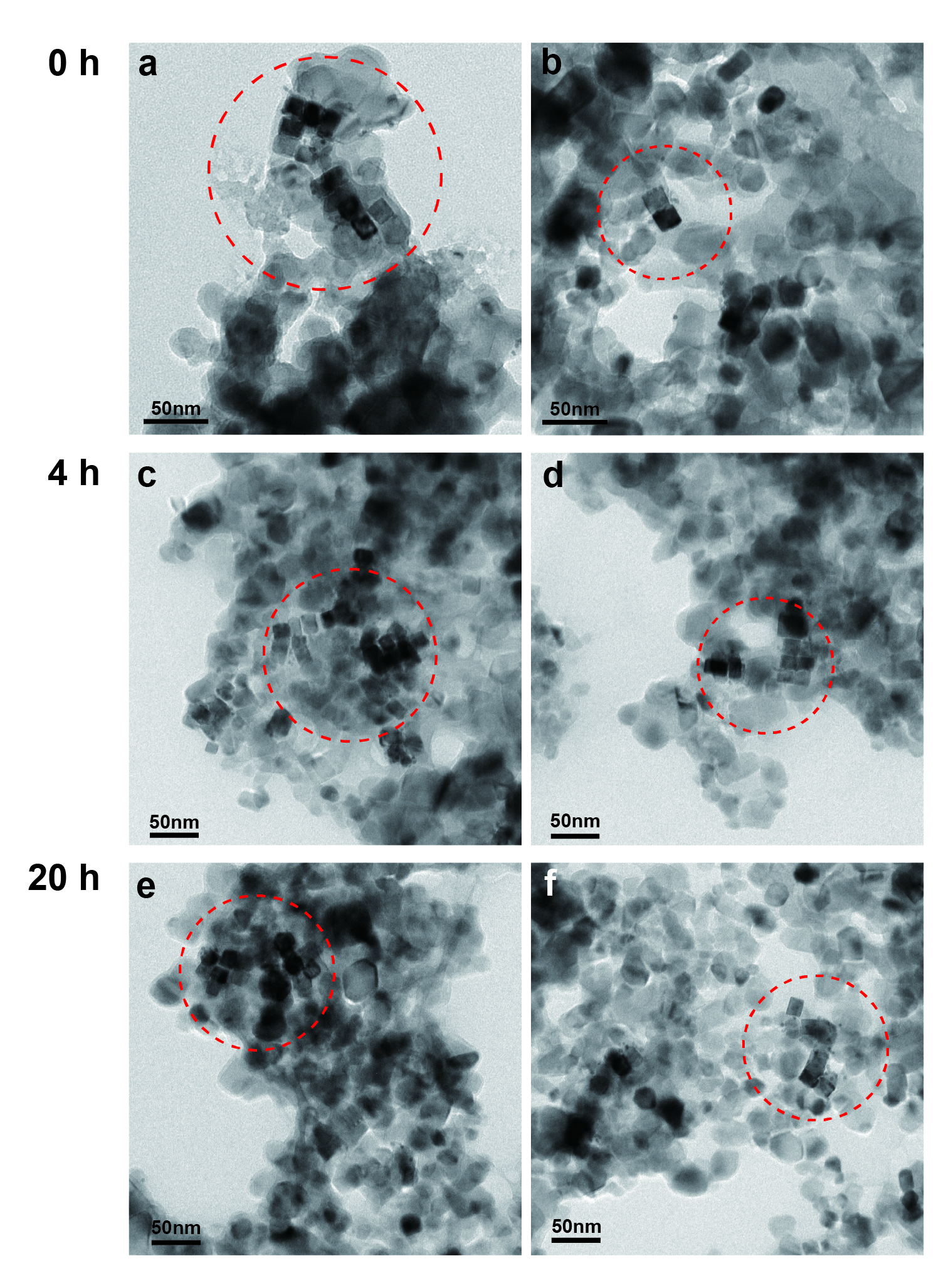


**Figure S8.** TEM images of the Pd@Pt_0.4_Pd_0.15_Ir_0.15_Ru_0.15_Rh_0.15-4L_ core-shell nanocubes/TiO_2_ after the photocatalytic hydrogen production test for a, b) 0 h, c, d) 4 h, and e, f) 20 h. The morphology of the Pd@Pt_0.4_Pd_0.15_Ir_0.15_Ru_0.15_Rh_0.15-4L_ core-shell nanocubes (highlighted with red circles) remained largely unchanged throughout the entire testing period. Additionally, the TiO_2_ nanoparticles retained their original morphology and exhibited nearly preserved circularity after 20 hours of illumination.


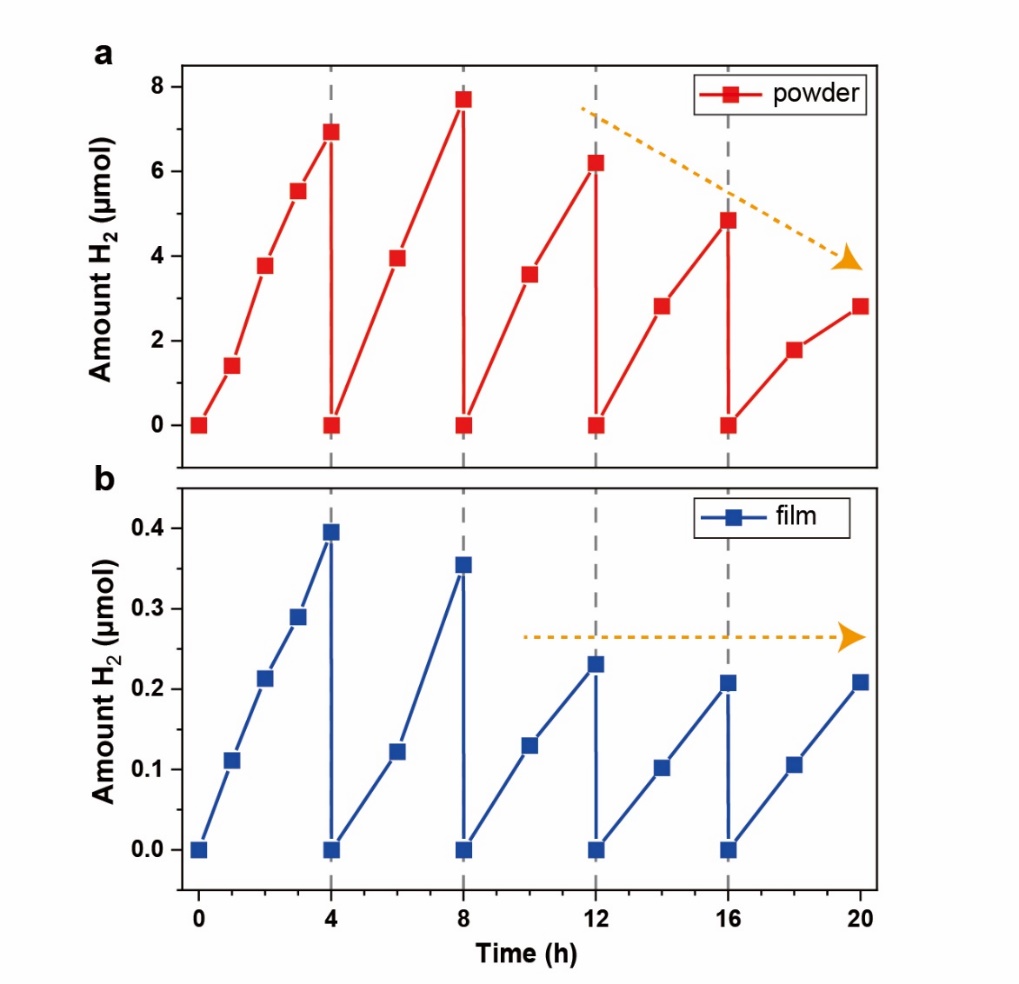


**Figure S9.** Long-term stability test for photocatalytic hydrogen production using a) powder dispersion and b) thin-film Pd@Pt_0.4_Pd_0.15_Ir_0.15_Ru_0.15_Rh_0.15-4L_ core-shell nanocubes over a 20-hour period. The stability assessment was conducted over five cycles, with each cycle lasting 4 hours under continuous light irradiation. Experimental conditions were identical to those described in the photocatalytic activity test in the Methods section. To ensure accurate measurements, the reactor was purged with Ar gas (5 L min^-1^ for 10 min) between cycles to remove residual H₂, after which it was resealed with a rubber stopper before resuming the next cycle.


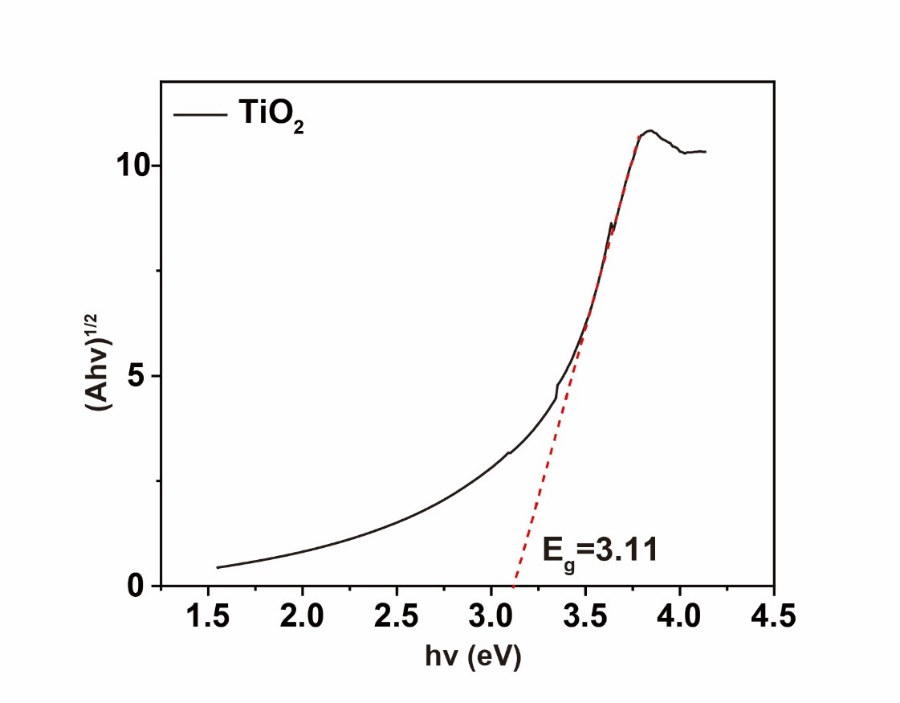


**Figure S10.** Tauc plot illustrating the determination of the band gap (E_g_) for TiO_2_ P25.


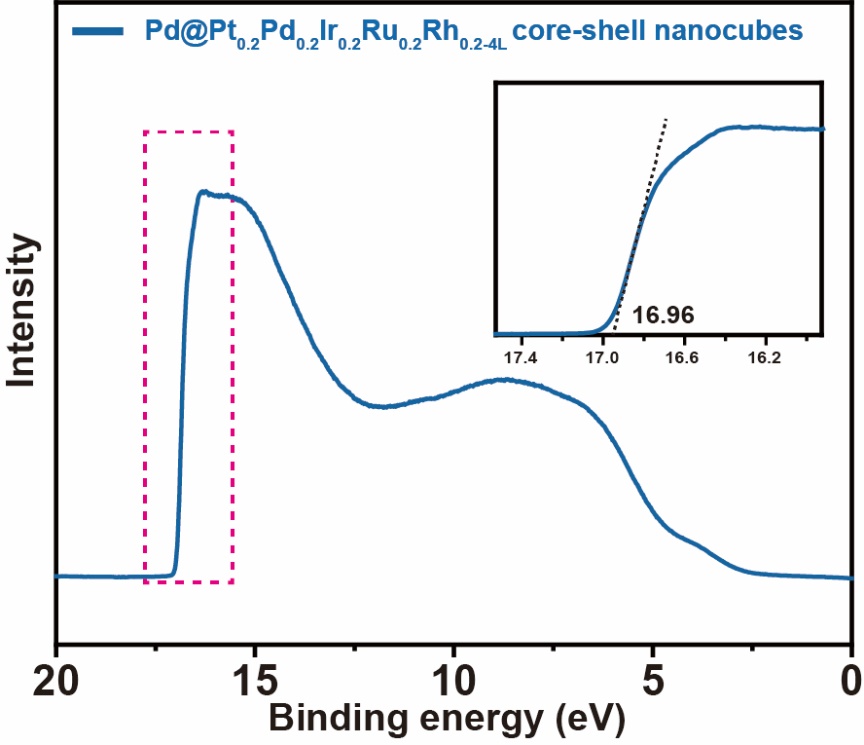


**Figure S11.** UPS spectra of Pd@Pt_0.2_Pd_0.2_Ir_0.2_Ru_0.2_Rh_0.2-4L_ core-shell nanocubes. The cut-off edge (inset: magnified view within the red frame) is used to determine the work function, which is calculated to be approximately 4.25 eV.

**
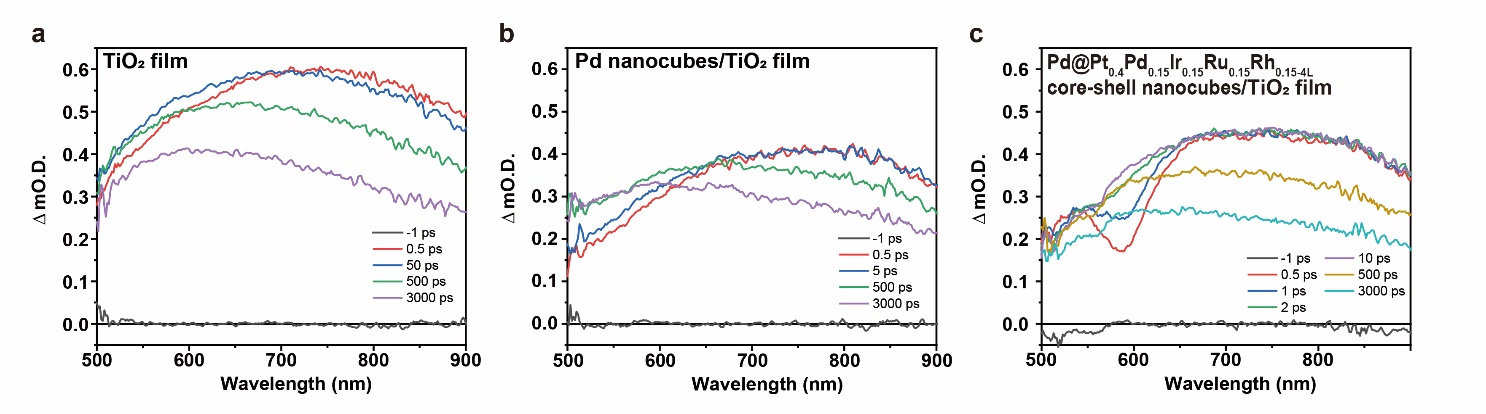
**

**Figure S12.** Ultrafast TA spectroscopy on ps-ns timescale was used to monitor the initial processes following the UV excitation at 355 nm for TiO_2_ films, Pd nanocubes/TiO_2_ films, and Pd@Pt_0.4_Pd_0.15_Ir_0.15_Ru_0.15_Rh_0.15-4L_ core-shell nanocubes/TiO_2_ films. Ps-ns TA spectra of a) TiO_2_ films, b) Pd nanocubes/TiO_2_ films, and c) Pd@Pt_0.4_Pd_0.15_Ir_0.15_Ru_0.15_Rh_0.15-4L_ core-shell nanocubes/TiO_2_ films following excitation wavelength at 355 nm (750 μW) at selected time delays under Ar atmosphere, respectively.


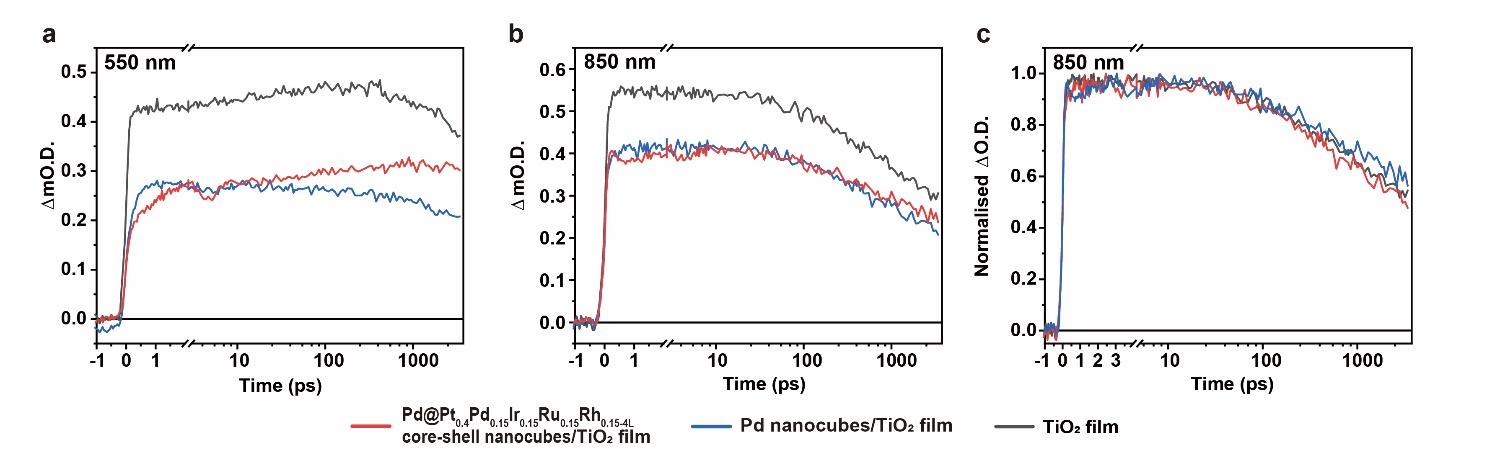


**Figure S13.** TA kinetic traces at a) 550 nm, and b) 850 nm of TiO_2_, Pd nanocubes/TiO_2,_ and Pd@Pt_0.4_Pd_0.15_Ir_0.15_Ru_0.15_Rh_0.15-4L_ core-shell nanocubes/TiO_2_ films following excitation wavelength at 355 nm (750 µW) under argon atmosphere on the ps–ns timescale, respectively. The signal at 550 nm corresponds to photoinduced absorption attributed to photogenerated holes in TiO_2_. As discussed in the main text, there is significant overlap of the SPR of the Pd nanoparticles across the visible region. c) The corresponding normalized kinetics at 850 nm, were used to compare the lifetime.


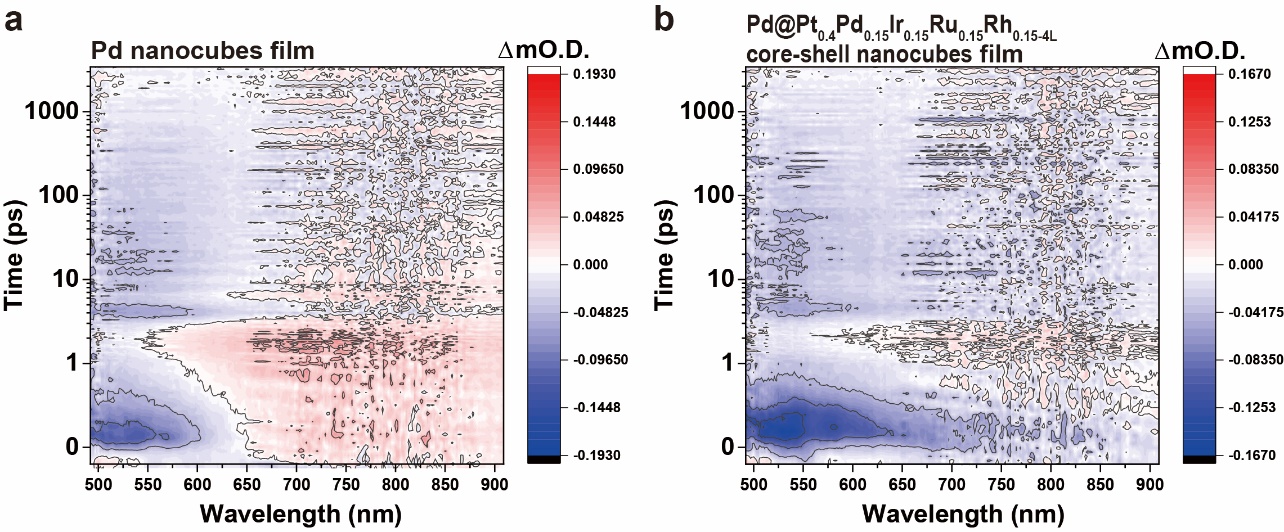


**Figure S14.** 2D color plot of TA spectra showing the plasmonic effect of a) Pd nanocubes film, and b) Pd@Pt_0.4_Pd_0.15_Ir_0.15_Ru_0.15_Rh_0.15-4L_ core-shell nanocubes films following excitation wavelength at 355 nm (750 µW) under argon atmosphere on the ps–ns timescale, respectively. a) and b) exhibit similar, the most pronounced contribution observed in the sample is from Pd nanocubes.


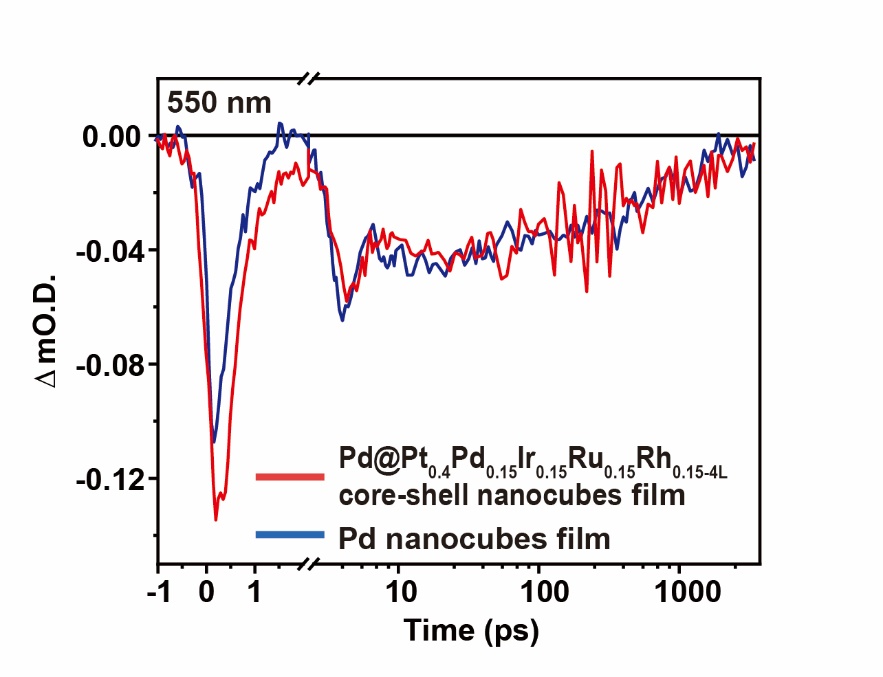


**Figure S15.** Single wavelength dynamics at 550 nm of Pd nanocube, and Pd@Pt_0.4_Pd_0.15_Ir_0.15_Ru_0.15_Rh_0.15-4L_ core-shell nanocubes films following excitation wavelength at 355 nm (750 µW) under Ar atmosphere on the fs–ns timescale. It shows that the plasmon mode of Pd nanocubes oscillating in frequency overlaps with the wavelength of the hole absorption of TiO_2_ in **Figure S12**.


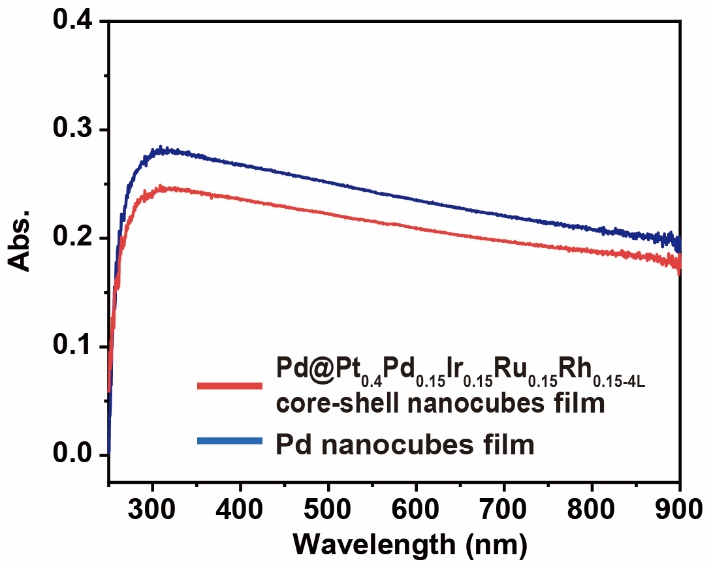


**Figure S16.** UV-vis spectra of Pd nanocubes films and Pd@Pt_0.4_Pd_0.15_Ir_0.15_Ru_0.15_Rh_0.15-4L_ core-shell nanocubes films. It shows the broad plasmon mode of Pd nanocubes, indicating that 355nm excitation used in the Pd nanocubes/TiO_2_ can also induce the Pd plasmon and the broad negative signal in **Figure S14** can be assigned to Pd plasmon bleach.


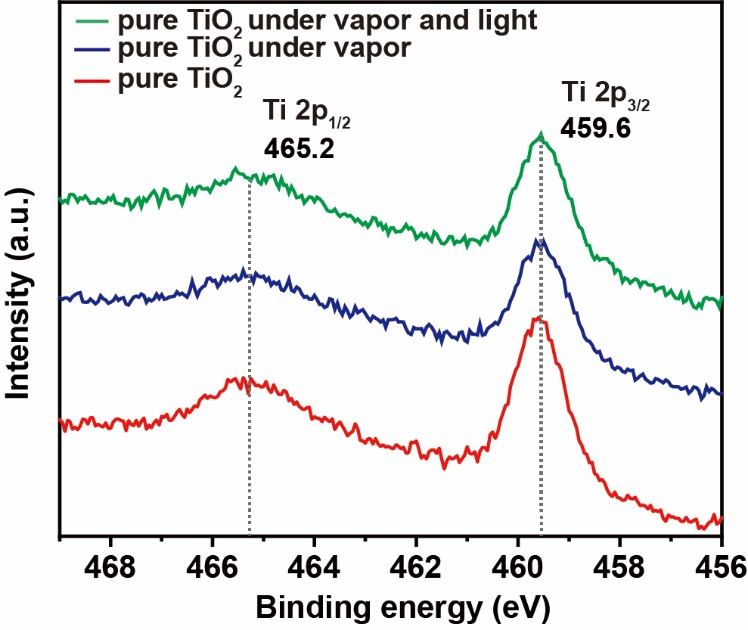


**Figure S17.** In situ XPS spectra of Ti 2p for pure TiO_2_ under water vapor and simulated sunlight (λ＞300 nm) irradiation. Despite the varying conditions during XPS measurement, the Ti 2p peak positions remained unchanged.


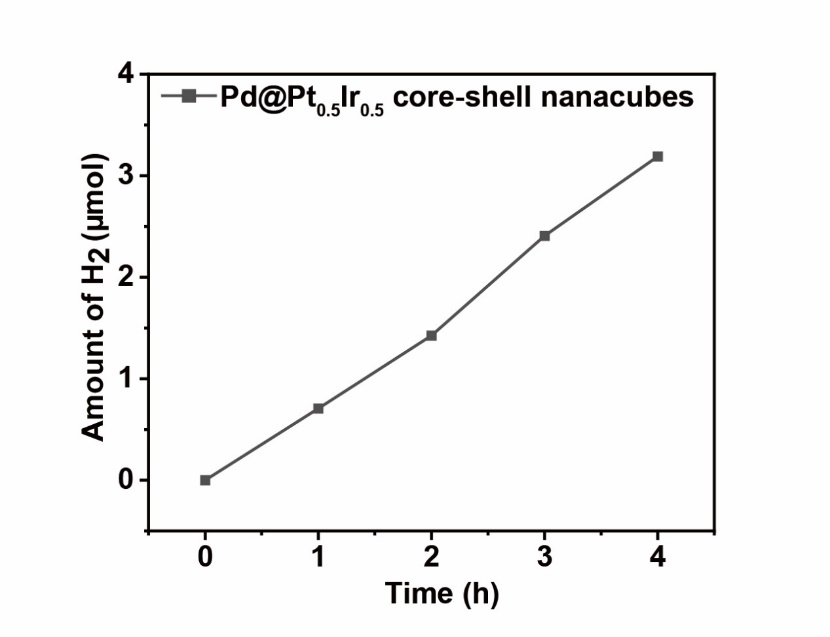


**Figure S18.** Photocatalytic hydrogen production performance of Pd@Pt_0.5_Ir_0.5_/TiO_2_ core-shell nanocubes, evaluated under the same conditions as other Pd@HEA_-4L_ core-shell nanocubes/TiO_2_. The average hydrogen production rate over 4 hours for Pd@Pt_0.5_Ir_0.5_ core-shell nanocubes/TiO_2_ is around 0.8 μmol h^-1^.


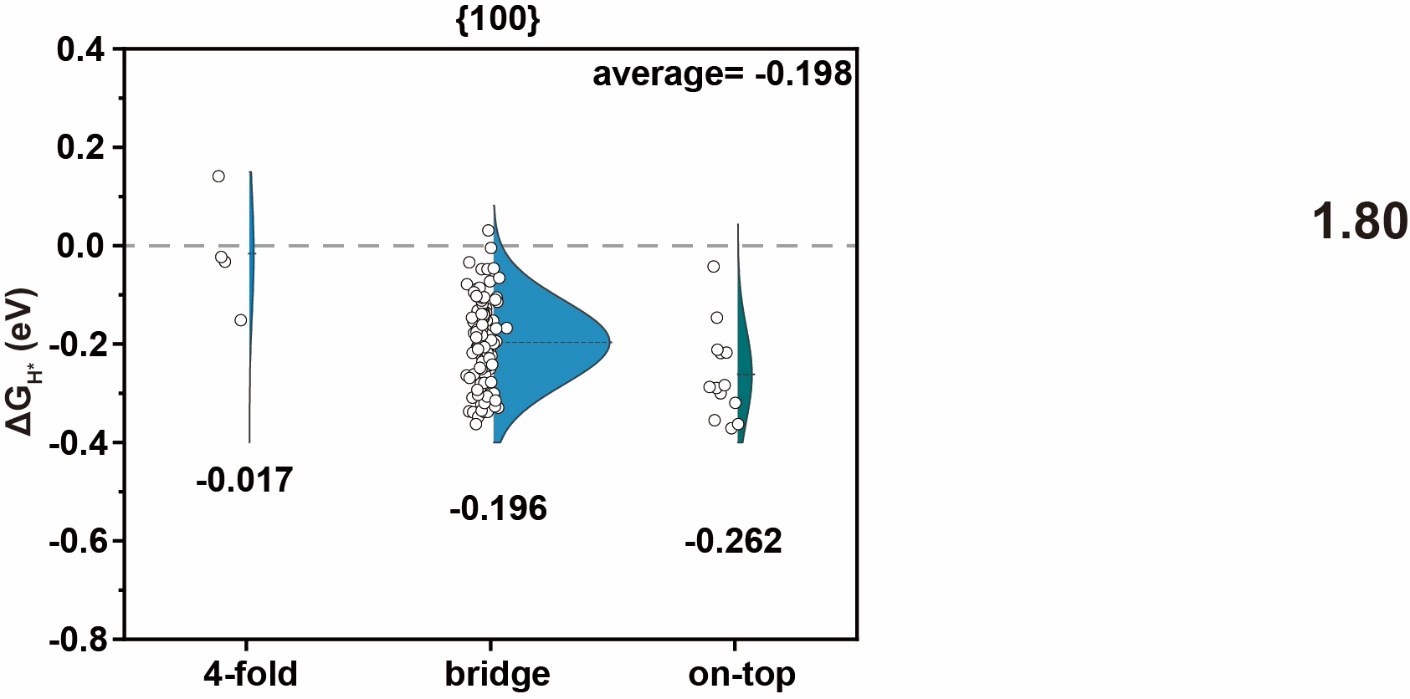


**Figure S19.** DFT analysis of [Pd@Pt_0.2_Pd_0.2_Ir_0.2_Ru_0.2_Rh_0.2_](mailto:Pd@Pt0.2Pd0.2Ir0.2Ru0.2Rh0.2-L), showing ΔG_H*_ values and their distributions across 4-fold, bridge, and on-top adsorption sites on the {100} facets. The average ΔG_H*_ value is determined to be -0.198 eV.


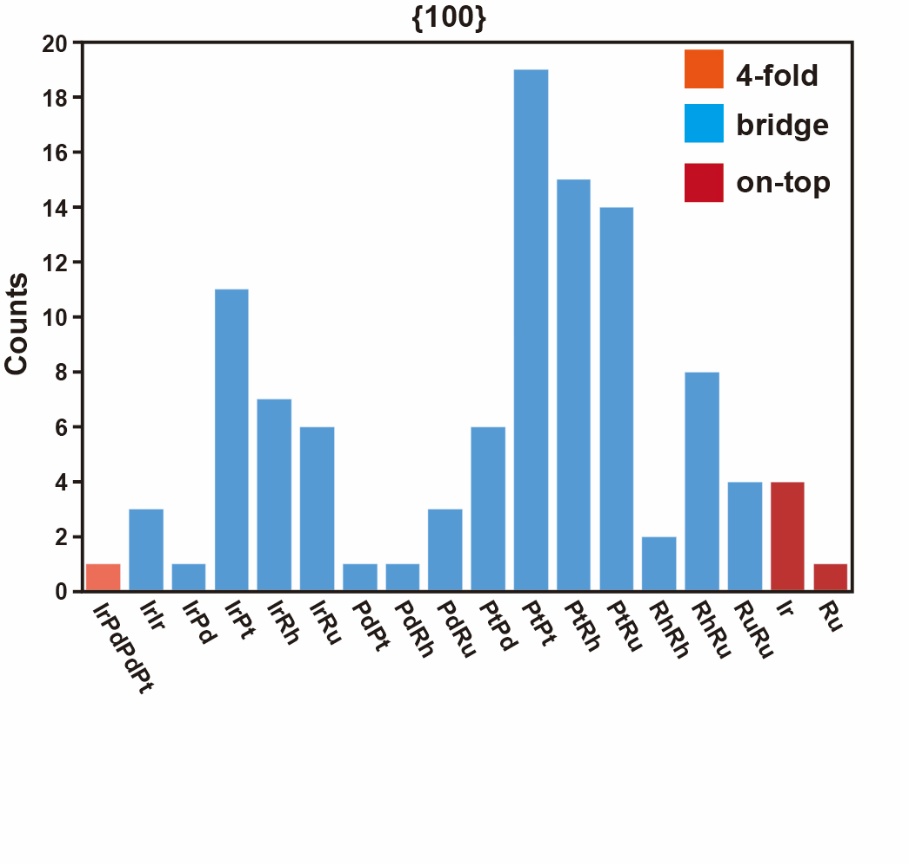


**Figure S20.** DFT analysis of Pd@Pt_0.4_Pd_0.15_Ir_0.15_Ru_0.15_Rh_0.15_, showing distributions of adsorption sites including 4-fold, bridge, and on-top sites on the {100} facets.

**Table S1.** Summary of high-entropy alloys used as co-catalysts for photocatalysis in recent

reports.

| Compositions | shape | Particle size [nm] | supports | Reaction | ref |
| --- | --- | --- | --- | --- | --- |
| FeCoNiCuMn | spherical nanoparticle | 3.5 | TiO_2_ | photocatalytic CO_2_ reduction | [1] |
| Pt_18_Ni_26_Fe_15_Co_14_Cu_27_ | spherical nanoparticle | 2.4 | protonated  g-C_3_N_4_ nanosheets | photocatalytic hydrogen production and simultaneous benzyl alcohol oxidation | [2] |
| PtCoFeRuMo | nanowire | diameter:  1.58 | WO_3_/ZnIn_2_S_4_ | photoelectrochemical autosensing of myoglobin | [3] |
| (Ni_40_Fe_30_Co_20_Al_10_)_90_Ti_10_ | nanoparticle | 10~30 | N/A | photocatalytic MB degradation | [4] |
| MnFeCoNiCu | nanoparticle | 15~85 | N/A | photocatalytic SMX, OFX and CFX degradation | [5] |
| Cd_0.9_Zn_1.2_Mn_0.4_Cu_1.8_Cr_1.2_Se_4.5_ | two-dimensional nanosheet | thickness:  2.5~3.5 | N/A | photocatalytic water splitting | [6] |
| FeMnCoTiV | powder | N/A | N/A | photocatalytic orange II dye degradation | [7] |
| CoNiCuPdRu | nanoparticle | 2.6 | TiO_2_ | photocatalytic CO_2_ hydrogenation | [8] |
| CuAgNiFeCoRuMn | nanoparticle | N/A | MgO | photocatalytic 4-nitrophenol hydrogenation | [9] |
| PtCuFeNiCo | nanoparticle | 4 | ZnCdS | photocatalytic water splitting | [10] |
| ZrNbTaMoW | powder | 1~1.5$\times$10^4^ | N/A | photocatalytic MB degradation | [11] |

**Table S2.** ICP-OES results of the atomic compositions of shells in the core-shell nanocrystals obtained and average atomic layers (*n*) of shell calculated in this work. The atomic percentage (at. %) of the shell was calculated from the atomic layers (*n*) and the size of Pd seeds with cubic and octahedral shapes.

| Sample name | Composition of shell [at. %] | | | | | Shell ratio derived from ICP-OES data [at. %] | Shell ratio derived from calculation [at. %] |
| --- | --- | --- | --- | --- | --- | --- | --- |
|  | Pd | Pt | Ir | Ru | Rh |  |  |
| Pd@Pd_0.25_Ir_0.25_Ru_0.25_Rh_0.25-4L_ core-shell nanocubes | 25.0 |  | 23.4 | 20.2 | 31.4 | 17.3 | 20.4 |
| Pd@Pt_0.2_Pd_0.2_Ir_0.2_Ru_0.2_Rh_0.2-4L_ core-shell nanocubes | 20.0 | 15.6 | 19.8 | 18.9 | 25.7 | 23.0 | 20.4 |
| Pd@Pt_0.4_Pd_0.15_Ir_0.15_Ru_0.15_Rh_0.15-4L_ core-shell nanocubes | 15.7 | 37.2 | 11.2 | 19.7 | 16.3 | 23.4 | 20.4 |
| Pd@Pt_0.6_Pd_0.1_Ir_0.1_Ru_0.1_Rh_0.1-4L_ core-shell nanocubes | 10.2 | 59.2 | 6.6 | 13.3 | 10.6 | 17.6 | 20.4 |
| Pd@Pt_0.8_Pd_0.05_Ir_0.05_Ru_0.05_Rh_0.05-4L_ core-shell nanocubes | 3.6 | 85.8 | 3.6 | 4.1 | 3.0 | 15.8 | 20.4 |
| Pd@Pt_-4L_  core-shell nanocubes |  | 100 |  |  |  | 16.2 | 20.4 |
| Pd@Pt_0.4_Pd_0.15_Ir_0.15_Ru_0.15_Rh_0.15-4L_ core-shell octahedra | 16.2 | 35.0 | 15.9 | 18.5 | 14.3 | 21.8 | 20.9 |

**Table S3.** Binding energies of each element in the XPS analysis (**Figure S5**) for TiO_2_, Pd@Pt_0.4_Pd_0.15_Ir_0.15_Ru_0.15_Rh_0.15-4L_ core-shell nanocubes, and Pd@Pt_0.4_Pd_0.15_Ir_0.15_Ru_0.15_Rh_0.15-4L_ core-shell nanocubes/TiO_2_ in different heat treatment stages.

| Sample | | Binding energy [eV] | | | | | | | |
| --- | --- | --- | --- | --- | --- | --- | --- | --- | --- |
|  |  | Pt 4f_7/2_ | Pd 3d_5/2_ | Ru 3d_5/2_ | Rh 3d_5/2_ | Ir 4f_3/2_ | Ti 2p_3/2_ | O 1s | C 1s |
| 1. | Pd@Pt_0.4_Pd_0.15_Ir_0.15_Ru_0.15_Rh_0.15-4L_ core-shell nanocubes | 72.5 | 336.8 | 281.1 | 308.5 | 61.8 |  |  | 286.5 |
| 2. | Pd@Pt_0.4_Pd_0.15_Ir_0.15_Ru_0.15_Rh_0.15-4L_ core-shell nanocubes /TiO_2_ after heat treatment at 190 ^o^C in air for 2 h | 71.8 | 335.9 | 280.6 | 307.7 | 61.2 | 459.8 | 531.0 | 286.6 |
| 3. | Pd@Pt_0.4_Pd_0.15_Ir_0.15_Ru_0.15_Rh_0.15-4L_ core-shell nanocubes /TiO_2_ after heat treatment at 190 ^o^C in air for 2 h and 10% H_2_/N_2_ for 1 h | 71.5 | 335.3 | 280.3 | 307.4 | 61.0 | 459.6 | 530.6 | 286.9 |
| Difference (2. – 1.) | | -0.7 | -0.9 | -0.5 | -0.8 | -0.6 |  |  |  |
| Difference (3. – 2.) | | -0.3 | -0.4 | -0.3 | -0.3 | -0.2 | -0.2 | -0.1 |  |
| Difference (3. – 1.) | | -1.0 | -1.3 | -0.8 | -1.1 | -1.8 |  |  |  |
| 4. | TiO_2_ after heat treatment at 190 ^o^C in air for 2 h |  | | | | | 459.4 | 530.6 | 286.8 |
| 5. | TiO_2_ after heat treatment at 190 ^o^C in air for 2 h and 10% H_2_/N_2_ for 1 h |  |  |  |  |  | 459.4 | 530.6 | 286.7 |

**Table S4.** Summary of carrier lifetime of metal/TiO_2_ composites analyzed by TAS analysis in recent reports.

| Sample | Solution | Reaction type | t_50%_ [ms] | Probed *λ* [nm] | Pumped *λ* [nm] | ref |
| --- | --- | --- | --- | --- | --- | --- |
| Pt/TiO_2_ | H_2_O, MeOH | water reduction with CB electrons | 2.2 | 1400 | 337 | [12] |
|  |  | the trapped electrons | 7 | 700 |  |  |
| Au/TiO_2_ | H_2_O, MeOH | oxidative coupling of methane | 3 | 950 | 355 | [13] |
| TiO_2_ PC-50 |  |  | 4.5$\times$10^-2^ |  |  |  |
| AgNW/TiO_2_ | H_2_O, MB | MB degradation | 2$\times$10^-9^ | 850 | 400 | [14] |
| Au_0.25_-Pd_0.25_/TiO_2_ | H_2_O, ACE | ACE degradation | 2.43$\times$10^-6^ | 510 | N/A | [15] |
| Au_0.25_-Pd_0.25_/TiO_2_ |  |  | 1.96$\times$10^-6^ |  |  |  |
| Au_0.5_/TiO_2_ |  |  | 1.51$\times$10^-6^ |  |  |  |
| CdS/TiO_2_ | H_2_O,  spiro-OMeTAD | electrons transport | 1$\times$10^-1^ | 650 | 450 | [16] |
| Sb_2_S_3_/TiO_2_ |  |  | 8$\times$10^-2^ |  |  |  |
| TiO_2_ P25 | D_2_O | the trapped holes | 3.6$\times$10^-9^ | 470 | 389 | [17] |
|  |  | the trapped electrons | 1.0$\times$10^-9^ |  |  |  |
|  |  |  |  | 600 |  |  |
| modified TiO_2_ film | H_2_O | the relaxed electrons | 2$\times$10^-3^ | 800 | 337 | [18] |

**Table S5.** Binding energies of each element in in situ XPS analysis (**Figure 5**) for Pd@Pt_0.4_Pd_0.15_Ir_0.15_Ru_0.15_Rh_0.15-4L_ core-shell nanocubes/TiO_2_ between different catalytic environments.

| Sample | | Binding energy [eV] | | | | | | |
| --- | --- | --- | --- | --- | --- | --- | --- | --- |
|  |  | Pt 4f_7/2_ | Pd 3d_5/2_ | Ru 3d_5/2_ | Rh 3d_5/2_ | Ir 4f_3/2_ | Ti 2p_3/2_ | O 1s  in TiO_2_ |
| 1. | Pd@Pt_0.4_Pd_0.15_Ir_0.15_Ru_0.15_Rh_0.15-4L_ core-shell nanocubes /TiO_2_ in vacuum | 71.6 | 335.5 | 280.2 | 307.3 | 61.0 | 459.7 | 530.7 |
| 2. | Pd@Pt_0.4_Pd_0.15_Ir_0.15_Ru_0.15_Rh_0.15-4L_ core-shell nanocubes /TiO_2_ under water vapor | 71.6 | 335.7 | 280.3 | 307.5 | 61.0 | 459.7 | 530.8 |
| 3. | Pd@Pt_0.4_Pd_0.15_Ir_0.15_Ru_0.15_Rh_0.15-4L_ core-shell nanocubes /TiO_2_ under water vapor and sunlight | 71.4 | 335.7 | 280.3 | 307.5 | 60.8 | 459.4 | 530.7 |
| Difference (2. – 1.) | | 0 | +0.2 | +0.1 | +0.2 | 0 | 0 | +0.1 |
| Difference (3. – 1.) | | -0.2 | +0.2 | +0.1 | +0.2 | -0.2 | -0.3 | 0 |
| Difference (3. – 2.) | | -0.2 | 0 | 0 | 0 | -0.2 | -0.3 | -0.1 |

Reference

[1] H. Huang, J. Zhao, H. Guo, B. Weng, H. Zhang, R. A. Saha, M. Zhang, F. Lai, Y. Zhou, R. Z. Juan, P. C. Chen, S. Wang, J. A. Steele, F. Zhong, T. Liu, J. Hofkens, Y. M. Zheng, J. Long, M. B. J. Roeffaers, *Adv. Mater.* **2024**, *36*, 2313209.

[2] L. Sun, W. Wang, P. Lu, Q. Liu, L. Wang, H. Tang, *Chin. J. Catal.* **2023**, *51*, 90.

[3] F. Xu, B. F. Xu, Q. Y. Ai, A. J. Wang, L. P. Mei, P. Song, J. J. Feng, *Chem. Eng. J.* **2024**, *489*, 151374.

[4] T. Wang, Y. Wang, N. Wang, S. Xu, Z. Han, Y. Wang, *Mater. Lett.* **2021**, *283*, 128817.

[5] S. Das, M. Sanjay, S. Kumar, S. Sarkar, C. S. Tiwary, S. Chowdhury, *Chem. Eng. J.* **2023**, *476*, 146719.

[6] J. Wang, Z. Wang, J. Zhang, S. Mamatkulov, K. Dai, O. Ruzimuradov, J. Low, *ACS Nano* **2024**, *18*, 20740.

[7] B. An, M. Yang, Y. Shang, S. Wang, C. Sun, K. Qian, X. Zou, Y. Shao, Q. Dong, C. Chu, F. Xue, C. Wang, J. Bai, *J. Mater. Chem. A* **2025**, *13*, 7999.

[8] H. Xiong, X. Ji, K. Mao, Y. Dong, L. Cai, A. Chen, Y. Chen, C. Hu, J. Ma, J. Wan, R. Long, L. Song, Y. Xiong, *Adv. Mater.* **2024**, *36*, 2409689.

[9] W. Al Zoubi, S. Leoni, B. Assfour, A. W. Allaf, J. H. Kang, Y. G. Ko, *InfoMat* **2024**, *7*, e12617.

[10] J. Wang, X. Niu, R. Wang, K. Zhang, X. Shi, H. Y. Yang, J. Ye, Y. Wu, *Appl. Catal. B: Environ.* **2025**, *362*, 124763.

[11] O. Zakir, O. Guler, R. Idouhli, A. Nayad, M. E. Khadiri, S. H. Guler, A. Abouelfida, B. Dikici, *J. Mater. Sci.* **2024**, *59*, 12050.

[12] H. Liu, M. Liu, R. Nakamura, Y. Tachibana, *Appl. Catal. B: Environ.* **2021**, *296*, 120226.

[13] X. Li, C. Li, Y. Xu, Q. Liu, M. Bahri, L. Zhang, N. D. Browning, A. J. Cowan, J. Tang, *Nat. Energy* **2023**, *8*, 1013.

[14] P. Makuła, M. Pacia, W. Macyk, *J. Phys. Chem. Lett.* **2018**, *9*, 6814.

[15] F. Wang, Y. Jiang, X. Wen, J. Xia, G. Sha, R. Amal, *ChemCatChem* **2013**, *5*, 3557.

[16] P. D. Tran, L. Xi, S. K. Batabyal, L. H. Wong, J. Barber, J. S. C. Loo, *Phys. Chem. Chem. Phys.* **2012**, 14, 11596.

[17] Y. Murakami, J. Nishino, T. Mesaki, Y. Nosaka, *Spectrosc. Lett.* **2011**, *44*, 88.

[18] X. Zhang, C. Bo, S. Cao, Z. Cheng, Z. Xiao, X. Liu, T. Tan, L. Piao, *J. Mater. Chem. A* **2022**, *10*, 24381.
